# Advances and Challenges in Low‐Temperature Upcycling of Waste Polyolefins via Tandem Catalysis

**DOI:** 10.1002/anie.202500559

**Published:** 2025-03-22

**Authors:** Wei Zhang, Sungmin Kim, Michele L. Sarazen, Mingyuan He, Jingguang G. Chen, Johannes A. Lercher

**Affiliations:** ^1^ State Key Laboratory of Petroleum Molecular & Process Engineering Shanghai Key Laboratory of Green Chemistry and Chemical Processes School of Chemistry and Molecular Engineering East China Normal University Shanghai 200062 China; ^2^ Institute of Eco‐Chongming 20 Cuiniao Road, Chenjia Town, Chongming District Shanghai 202162 China; ^3^ Institute for Integrated Catalysis Pacific Northwest National Laboratory Richland WA 99354 USA; ^4^ Department of Chemical and Biological Engineering Princeton University Princeton NJ 08544 USA; ^5^ Department of Chemical Engineering Columbia University New York NY 10027 USA; ^6^ Department of Chemistry and Catalysis Research Center Technical University of Munich Lichtenbergstrasse 4 Garching 85747 Germany

**Keywords:** C─C cleavage, Exothermic reaction, Mild conditions, Polyolefin upcycling, Tandem catalysis

## Abstract

Polyolefin waste is the largest polymer waste stream that could potentially serve as an advantageous hydrocarbon feedstock. Upcycling polyolefins poses significant challenges due to their inherent kinetic and thermodynamic stability. Traditional methods, such as thermal and catalytic cracking, are straightforward but require temperatures exceeding 400 °C for complete conversion because of thermodynamic constraints. We summarize and critically compare recent advances in upgrading spent polyolefins and model reactants via kinetic (and thermodynamic) coupling of the endothermic C─C bond cleavage of polyolefins with exothermic reactions including hydrogenation, hydrogenolysis, metathesis, cyclization, oxidation, and alkylation. These approaches enable complete conversion to desired products at low temperatures (<300 °C). The goal is to identify challenges and possible pathways for catalytic conversions that minimize energy and carbon footprints.

## Graphical abstract

This review presents an overview of recent advances in mild catalytic transformations of polyolefins, emphasizing the kinetic and thermodynamic interplay between endothermic C─C bond cleavages and the exothermic formation of new C─H, C─C, and C─O bonds. The aim is to identify existing challenges and potential routes for catalytic conversions that reduce energy consumption and carbon footprints.

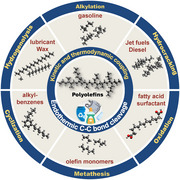

## 1 Introduction

Polyolefins comprised of polyethylene (PE) and polypropylene (PP) emerge as the primary category of synthetic plastics, with current demand estimated at over 200 million tons worldwide, constituting nearly two‐thirds of all plastics consumption.^[^ ^1^, ^2^ ^]^ The global polyolefin market is anticipated to sustain its growth in the forthcoming years. In 2023 alone, the new capacity of PE and PP was 17.5 million tons.^[^ ^3^ ^]^ Due to their low cost, light weight, versatility, and durability, polyolefins have become ubiquitous in our daily lives.^[^ ^4^ ^]^ Their extensive utility includes single‐use items such as plastic bags, packaging materials, and disposable masks, as well as diverse manufacturing sectors encompassing automotive, construction, pharmaceuticals, medical, electronics, and electricals.^[^ ^5^ ^]^ However, their excessive consumption presents a persistent risk of severe environmental pollution due to their high resistance to degradation, resulting in their accumulation in landfills and uncontrolled release into the environment.^[^ ^6^, ^7^ ^]^

As the largest polymer waste stream, discarded polyolefins can be effectively separated from other plastics. Their substantial presence represents a notable yet untapped hydrocarbon resource.^[^ ^8^, ^9^ ^]^ In 2019, the United States generated an estimated 30 million metric tons of polyolefin waste annually, equivalent to 1.2 million barrels per day (bbl/d) of refining capacity. This amount matches the output of two of the largest U.S. refineries and accounts for approximately 7% of the nation's total refining capacity.^[^ ^10^ ^]^ Considering the potential for co‐processing, upcycling polyolefins as a clean hydrocarbon feedstock offers substantial benefits,^[^ ^11^, ^12^ ^]^ obviating the necessity for saturation and heteroatom removal.^[^ ^13^, ^14^ ^]^ The deconstruction of polyolefin waste allows for the production of a diverse range of products, including chemicals, intermediates, fuels, and lubricants suitable for existing end markets. These can either be employed directly as end‐use products or as feedstocks for well‐targeted processes. Consequently, polyolefin upcycling emerges as a rapidly evolving research domain, garnering significant attention in the global pursuit of carbon neutrality and a circular economy.

**Figure 1:**
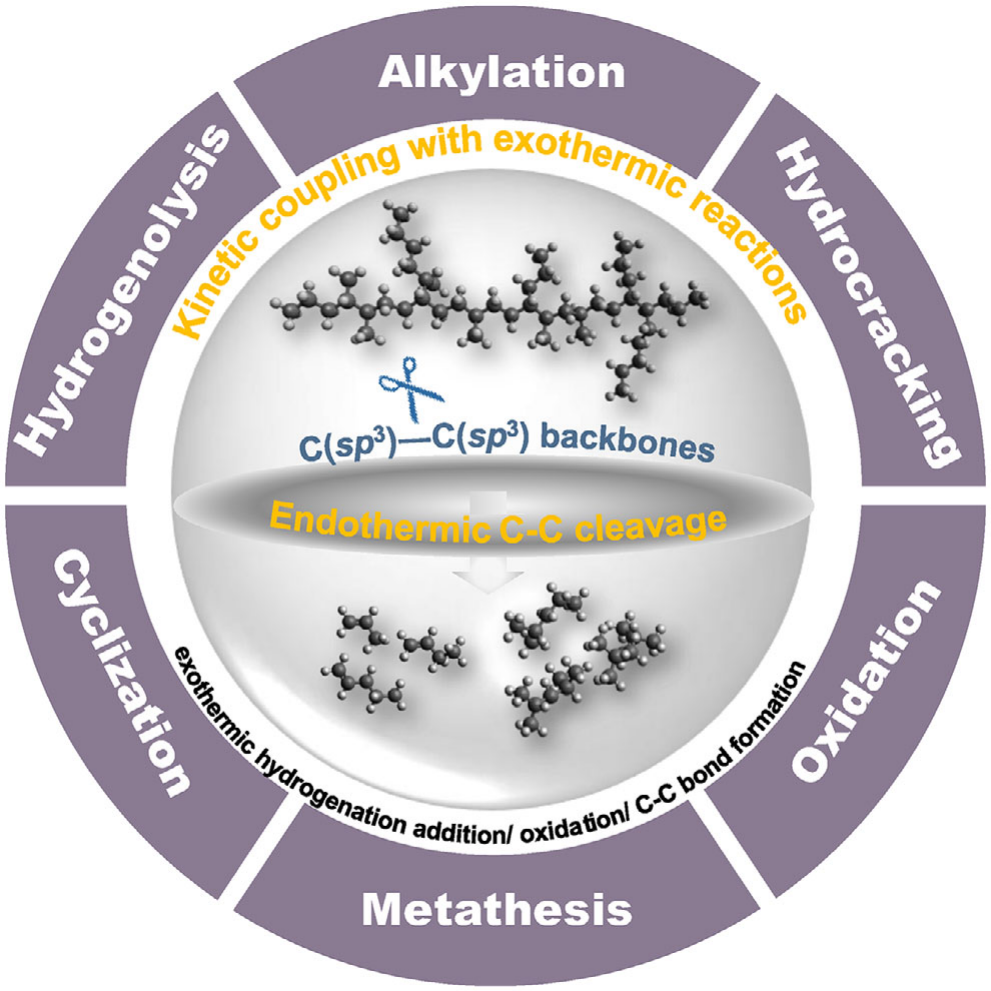
A summary of recent advances in low‐temperature upcycling strategies for waste polyolefins emphasizes the kinetic coupling of endothermic C─C bond cleavage in polyolefins with various exothermic reactions. These include hydrogenation, hydrogenolysis, metathesis, cyclization, oxidation, and alkylation, which process the alkene intermediates formed in the primary cleavage.

While polyolefins offer potential, their kinetic and thermodynamic stability, caused by the highly stable C(*sp* ^3^)─C(*sp* ^3^) and C(*sp* ^3^)─H bonds, presents a significant barrier to the conversion at modest conditions.^[^ ^15^ ^]^ These bonds are significantly more stable than the carbon‐heteroatom bonds in functionalized polymers such as polyethylene terephthalate, polyesters, and polyamides.^[^ ^16^ ^]^ Traditional methods, such as thermal and catalytic cracking, are straightforward but necessitate temperatures exceeding 400 °C to overcome equilibrium limitations in the endothermic C─C and C─H bond cleavage.^[^ ^17^, ^18^ ^]^ Alternatively, recent catalytic techniques have emerged in lowering reaction temperatures via kinetic coupling of the endothermic C─C cleavage within polyolefins with exothermic hydrogen addition and C─C bond formation (Figure 1). The selected reactions utilize a variety of co‐reactants, including H_2_, light paraffins, olefins, and O_2_. They serve to balance the energy required for breaking a C─C bond with the formation of a new one, either through metathesis or oxidation or via the addition of hydrogen, which can take the form of H_2_ (hydrogenolysis and hydrocracking) or R─H (alkylation). Examples employing reactions such as hydrogenolysis,^[^ ^19^, ^20^, ^21^, ^22^, ^23^, ^24^, ^25^, ^26^ ^]^ hydrocracking,^[^ ^27^, ^28^, ^29^ ^]^ cyclization,^[^ ^30^, ^31^, ^32^ ^]^ metathesis,^[^ ^33^, ^34^, ^35^, ^36^, ^37^ ^]^ oxidation,^[^ ^38^, ^39^ ^]^ and alkylation^[^ ^40^ ^]^ have achieved conversions that surpass the equilibrium limits of standalone cracking processes. In principle, these mild catalytic transformations with polyolefins share similarities with those employed in the conversion of smaller hydrocarbon molecules in traditional refining operations.^[^ ^41^, ^42^, ^43^, ^44^, ^45^, ^46^ ^]^ Fundamental insights from previously established strategies with gaseous and liquid alkanes can serve as a foundation for comprehending polyolefin conversion.

While several reviews have either summarized progress in these catalytic systems or discussed the potential of polyolefin as an untapped resource for producing new functional polymeric materials and valuable feedstock,^[^ ^14^, ^15^, ^16^, ^47^, ^48^, ^49^, ^50^, ^51^, ^52^, ^53^, ^54^, ^55^, ^56^, ^57^, ^58^, ^59^, ^60^ ^]^ few of them have focused on the chemically more challenging polyolefins.^[^ ^61^, ^62^, ^63^, ^64^ ^]^ The rapidly expanding field of polyolefin upcycling is expanding rapidly but still lacks a comprehensive understanding of how to selectively and efficiently upgrade polyolefins. This review presents an overview of the latest advances in mild catalytic transformations of polyolefins,^[^ ^65^ ^]^ emphasizing the principle of low‐temperature catalytic upcycling. In particular, it highlights the kinetic and thermodynamic interplay between endothermic C─C bond cleavages and the exothermic formation of new C─H and C─C bonds. The aim is to identify existing challenges and potential routes for catalytic conversions that reduce energy consumption and carbon footprint.

## 2 Thermodynamics and Kinetics of Catalytic Upcycling of Polyolefins

In polymer science, thermodynamic control of polymerization and depolymerization can theoretically enable a reversible transition between polymers and monomers, with the ceiling temperature (*T*_C_) marking the de/polymerization equilibrium point. Above *T*_C_, depolymerization is favored thermodynamically over polymerization. Given the stable saturated C─C and C─H bonds in polyolefins, their *T*_C_ surpasses 400 °C. As a result, techniques such as direct pyrolysis and cracking into light olefins require temperatures considerably higher than *T*_C_.^[^ ^52^, ^66^ ^]^ However, these methods lack control in product distribution and can kinetically favor unwanted side reactions with lower activation energies, yielding lower‐value mixtures such as gases, waxes, and char.^[^ ^58^ ^]^

**Figure 2:**
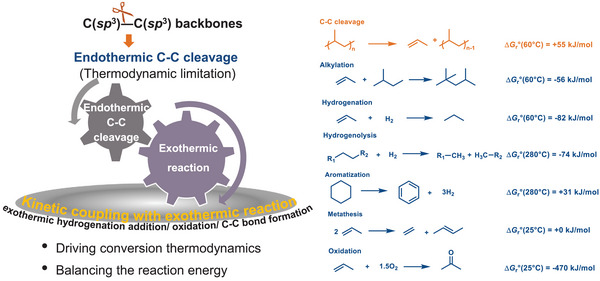
The thermodynamic analysis shows model reactions with respective Gibbs free energies using HSC Chemistry 10 software at the indicated temperatures. The unfavorable thermodynamics of the initial C─C cleavage at low temperatures need to be offset mainly by alkylation and hydrogen addition (hydrogenation and hydrogenolysis) and oxidation, and near‐neutral isomerization and metathesis.

From a thermodynamic perspective, the entropy change (Δ*S*) in the depolymerization process is invariably positive, leading to a decrease in the overall reaction free energy (Δ*G* = Δ*H* − TΔ*S*). This tendency favors a broad distribution of small molecules, especially at high temperatures. To achieve the desired selectivity, the depolymerization process should be operated at low temperatures under kinetic control, tailored through the design of catalysts and reaction systems.^[^ ^15^ ^]^ For example, hydrogenolytic C─C cleavage operates at temperatures lower than in pyrolytic processes, which allows for better control on product distribution and demonstrates the potential for catalytic upcycling of polyolefin waste. The enthalpy change (Δ*H*), dependent on the target products, is fixed and remains unaffected by catalysts. Polyolefin depolymerization faces significant enthalpic stability challenges. To overcome the thermodynamic constraints at lower reaction temperatures, the endothermic C─C cleavage must be kinetically coupled to exothermic reactions. Figure 2 highlights recent advancements in catalytic strategies, illustrating that the unfavorable thermodynamics of C─C cleavage at low temperatures can be offset by exothermic reactions such as hydrogenation, hydrogenolysis, metathesis, cyclization, oxidation, and alkylation. The chosen reactions either balance the energy of breaking a C─C bond with that of forming a new one (as in metathesis and oxidation) or through the addition of hydrogen, either in the form of H_2_ (during hydrogenation and hydrogenolysis) or R─H (in alkylation).

While thermodynamics establishes the viability of coupled reactions, kinetics determines their rates and product profiles. Achieving high conversion rates at lower temperatures requires kinetic control, especially in coupling strategies within cooperative catalysis. Even though the catalytic mechanisms of individual reaction steps are well understood for converting small hydrocarbons, lowering the activation energy to achieve kinetic control remains a significant challenge in polyolefin deconstruction. It should be noted that C─C bond cleavage in long polyolefin chains is more daunting than in small paraffins, primarily due to the intrinsic difficulty of polymers in chain mobility and access to catalytic sites. In particular, in tandem catalysis with diverse catalytic functions, the distance between various site types can impede the interaction of polymer strands with active catalytic sites, thereby retarding the reaction.

Catalyst selection and reaction conditions are pivotal for both thermodynamic and kinetic control. The breakdown of polyolefins into specific products is mainly governed by enthalpy changes (Δ*H*) and a specific reaction barrier (*E*_a_). Namely, Δ*H* determines the thermodynamics of coupling reactions, whereas *E*_a_, manipulated kinetically via catalysis, addresses the kinetic limitations by enabling targeted reactions to proceed at significant rates.

As such, we will provide a comprehensive overview and critical assessment of recent advances in the upgrading of polyolefins and model reactants. This encompasses the kinetic and thermodynamic coupling between endothermic C─C bond cleavages and exothermic processes, including hydrogenation, hydrogenolysis, metathesis, cyclization, oxidation, and alkylation, aiming to establish proof‐of‐concept demonstrations at lower temperatures, targeting single‐stage, energy‐equilibrated conversion processes that yield diverse target products.

## 3 Recent Advances in Catalytic Upcycling of Polyolefins

### 3.1 Coupling C─C Cleavage with Alkylation

Alkylation, a widely used conversion process in petroleum refineries, produces highly branched gasoline‐range hydrocarbons from upgrading low molecular‐weight alkenes and isoparaffins, which are byproducts during fluid catalytic cracking (FCC).^[^ ^43^ ^]^ Typically, this involves the alkylation of isobutane (iC_4_) with C_3–5_ alkenes in the presence of strong acids and leads to the formation of complex mixtures of branched alkanes, called alkylate, that are blended to improve octane number.^[^ ^67^ ^]^ Despite the increasing influence of battery‐powered electric vehicles on the petrochemical market, the global alkylation market is poised for significant growth in the years to come.^[^ ^68^ ^]^ This growth is driven by geopolitical factors and the sustained demand for high‐octane gasoline, which remains essential as long as automobiles continue to rely on it as a primary fuel source (i.e., hybrid vehicles).^[^ ^69^ ^]^

Polyolefin waste holds potential as an advantageous feedstock for a next‐generation refinery, serving as a replacement for the C3–C5 olefin cut to produce alkylates. Conceptually, the endothermic cleavage of C─C bonds is kinetically coupled with the exothermic alkylation of isoparaffins. This coupling becomes thermodynamically favorable by the formation of the new C─C bond between the olefins and the hydride transfer‐activated hydrogen‐rich alkane containing a tertiary carbon atom (e.g., iC_4_ and iC_5_).

**Figure 3:**
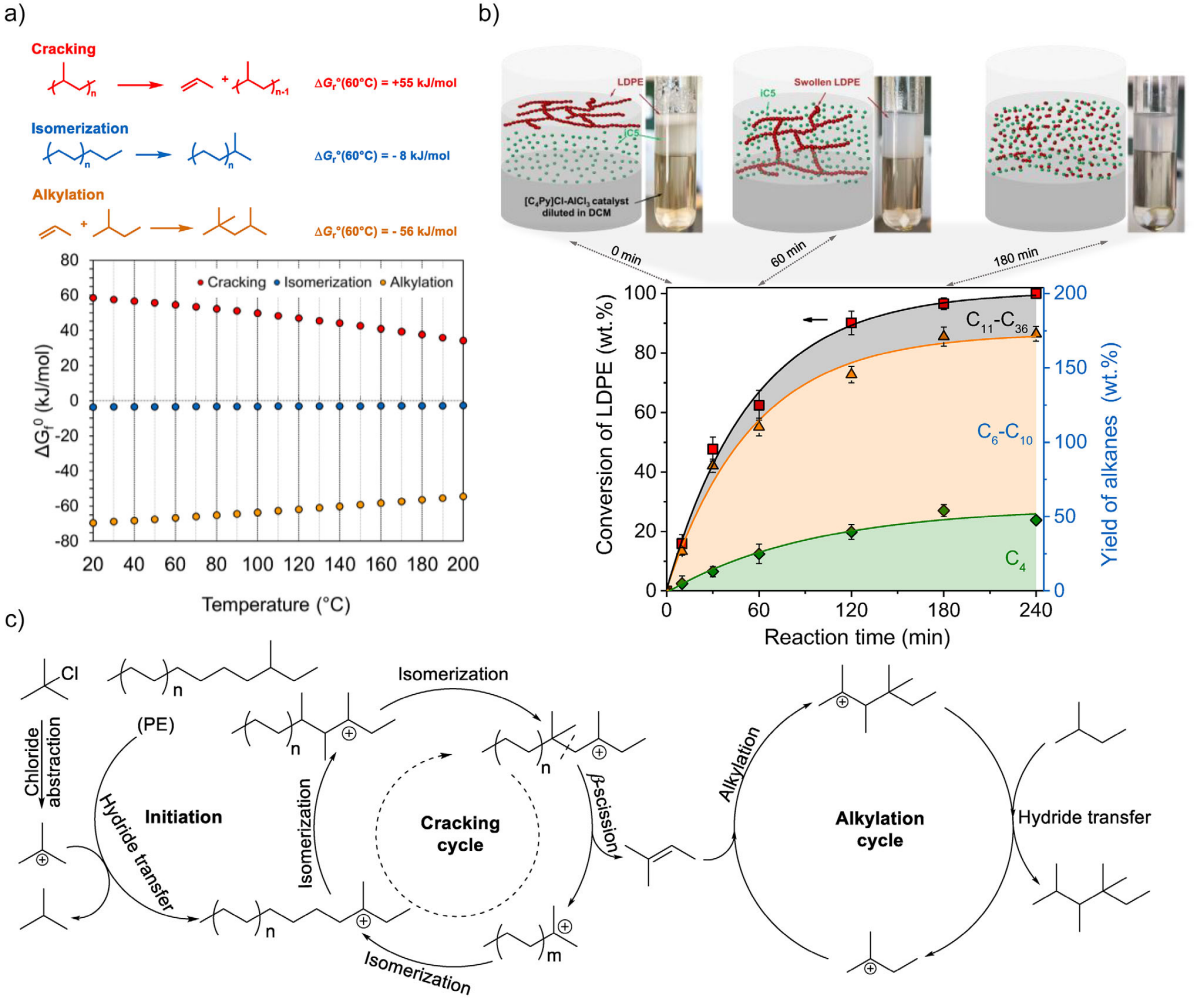
Tandem cracking for converting PE into liquid isoalkanes. a) Thermodynamic calculation of model reactions for cracking, alkylation, and isomerization with the respective Gibbs free energy of reaction at varying temperatures. b) Process depiction of the one‐pot catalytic LDPE/iC_5_ upcycling into liquid alkanes over Lewis acidic chloroaluminate ionic liquid at 70 °C. The time‐resolved conversion profile of LDPE and cumulative yield of alkanes (C_4_, green diamond; C_6_–C_10_, orange triangles; C_11_–C_36_, red squares). Reaction conditions were as follows: LDPE, 200 mg; iC_5_, 800 mg; [C_4_Py]Cl‐AlCl_3_ ([C_4_Py]Cl–AlCl_3_ molar ratio of 1:2), 3 mmol; TBC as an additive, 0.05 mmol (5 mg); DCM 3 mL, 70 °C. The snapshots of the LDPE conversion (top) are at 0, 60, and 180 min from left to right. Curves represent the optimal fit to the data, and all data were repeated at least five times and are shown as mean data points with error bars. c) Proposed reaction mechanism for the tandem cracking‐alkylation process of a polyolefin with iC_5_. The 2‐methyl‐butene formed in the cracking cycle is depicted as an example. Reprinted with permission from Ref. [40]. Copyright 2023, AAAS.

Thermodynamic analyses (see Figure 3a) indicate that olefin alkylation with isoparaffin is thermodynamically favored under mild conditions, with a Gibbs free energy change (∆*G*°) of approximately −56 kJ mol^−1^ at 60 °C and ambient pressure. This will overcome the thermodynamic limitations of the endothermic cleavage of C─C bonds. The exergonic nature of isomerization, with a ∆*G*° of around −8 kJ mol^−1^, aids to that Gibbs free energy change. Importantly, acid‐catalyzed cracking and alkylation are posited to share carbenium ions as intermediates,^[^ ^43^ ^]^ enabling them to occur simultaneously within the same reaction medium and with the same catalyst.

Our group used this tandem cracking‐alkylation strategy for transforming discarded polyolefins into gasoline‐range isoalkanes using a single‐stage process at temperatures below 100 °C in a few hours (Figure 3b).^[^ ^40^ ^]^ The mass yield of alkanes observed is approximately twice the mass of LDPE converted within 3 h. Approximately 2/3 of the products were in the gasoline/kerosene range, somewhat less than 1/3 in the form of isobutane (iC_4_). The negligible production of C_1_–C_3_ hydrocarbons minimizes carbon loss. All light isoparaffins (C_4_–C_6_) produced during the process, along with any unreacted isopentane, can serve as alkylation partners. This setup enables largely self‐sufficient operation and facilitates the complete conversion of recycled materials. The acidic chloroaluminate‐based ionic liquids (e.g., *n*‐butyl pyridinium chloride‐aluminum chloride) catalyze both the decomposition of polyolefins and the alkylation with light alkanes. Besides its function of generating the active sites,^[^ ^70^ ^]^ the presence of a high concentration of ions in the ionic liquid is critical for the high conversion rate of the polyolefin at such low temperatures; it not only stabilizes carbenium ions that are involved in all transition states of the two cycles but also allows for easy separation of nonpolar alkane products from the reaction media. The proposed mechanism (Figure 3c) suggests two interrelated catalytic cycles based on carbenium ions, independently generated within polymer strands and iC_5_, necessitating a Lewis acid‐catalyzed hydride transfer. Cracking‐derived alkenes act as intermediates that link these cycles. The process is initiated by small amounts of *tert*‐butyl chloride, providing the initial carbenium ions for the chain process.^[^ ^71^ ^]^ These carbenium ions preferentially abstract a hydride from tertiary carbon atoms, both from the polymer and from iC_5_. The carbenium ions formed in the polymer strands tend to undergo skeletal isomerization and cracking via β‐scission. Simultaneously, alkenes formed in this process (the cracking cycle) react with carbenium ions formed from iC_5_ in the alkylation cycle. Larger fragments undergo further cracking and alkylation cycles to the branched alkylate.

We further found that Lewis acid chlorides, especially aluminum and gallium chlorides, showed greater activity than the corresponding ionic liquids and did not require the addition of *tert*‐butyl chloride as the carbenium ion initiator.^[^ ^72^ ^]^ Notably, at 60 °C, anhydrous AlCl_3_ achieved full LDPE conversion with over 70% selectivity for liquid alkanes in the gasoline range, significantly surpassing other Lewis acid catalysts in efficiency by two orders of magnitude. This reactivity is attributed to the Lewis acid's ability to facilitate chloride and hydride transfer and to stabilize carbenium ions in solution, rather than to its acid strength. The Lewis acid and dichloromethane solvent create a highly polar environment that leads to the formation of an electron donor‐acceptor complex (i.e., AlCl_3_ ← ClCH_2_Cl). It further evolves into a chloromethyl‐carbenium ion and AlCl_4_^−^ pair, initiating carbenium ion chemistry. Intermolecular hydride transfer is then critical for propagating the carbenium ions in the cracking and alkylation cycles along with concurrent isomerization. Remarkably, Al^3+^ is identified as the most effective catalyst for this elementary step, with the softer and more polarizable nature of the halogenide enhancing the hydride transfer rate.

Currently, industrial alkylation predominantly uses concentrated sulfuric acid and hydrogen fluoride as catalysts for producing branched gasoline‐range alkanes from light isoparaffins (isobutane/isopentane) and C_3_–C_5_ olefins. The alkylation typically needs a large excess of isoparaffins over the olefins (*n*_isoparaffins_–*n*_olefins_ = 7/10) to lower the competing oligomerization of olefins to acid‐soluble oils (ASO) as by‐products. The synchronous release of olefins via polyolefin cracking in the presented cascade cracking‐alkylation conceptually allows for better control on product distribution and minimizes the formation of the “red‐oil” waste,^[^ ^67^ ^]^ making polyolefins a potential feed for alkylation in existing refineries.

### 3.2 Catalytic Hydroconversion via Hydrogenolysis and Hydrocracking

Catalytic hydroconversion, which includes hydrogenolysis and hydrocracking, has arguably emerged as the most prevalent strategy for converting polyolefins into hydrocarbons within specific molecular weight ranges like fuels, lubricants, and waxes at moderate temperatures (200–350 °C).^[^ ^73^, ^74^ ^]^ Hydrogenolysis and hydrocracking are both exothermic catalytic processes involving the addition of H_2_ that involve hydrogen addition to alkenes resulting from endothermic C─C bond cleavage at relatively low temperatures, enabling significant advances in energy efficiency. Despite their similarities, they have distinct mechanistic pathways and end products, as well as varied catalysts. Hydrogenolysis involves C─C bond cleavage followed by hydrogenation on metal surfaces, producing smaller molecules that incorporate hydrogen into the newly formed fragments. By contrast, hydrocracking employs bifunctional cooperative catalysts, combining metal‐induced C─H bond activation with acid‐catalyzed C─C cleavage and subsequent skeletal rearrangements, yielding more valuable products such as gasoline, diesel, and other fuels.^[^ ^75^ ^]^ Notably, hydrogenolysis typically operates at lower temperatures between 200–250 °C, yielding significant amounts of gaseous products (predominantly methane), but produces less gasoline range alkane than hydrocracking, which operates within the 250–375 °C range.^[^ ^73^ ^]^ Due to the elevated temperatures used in hydrocracking via carbenium ion chemistry, C─C cleavage occurs more rapidly than in metal‐catalyzed hydrogenolysis. In general, hydrocracking predominantly produces branched (liquid) alkanes, while hydrogenolysis tends to produce gaseous and heavier straight‐chain alkanes. Moreover, significant methane production distinctly characterizes hydrogenolysis, whereas hydrocracking typically avoids C1 intermediates like methyl cations.

#### 3.2.1 Hydrogenolysis

In petroleum refineries, hydrogenolysis is often considered an undesired side reaction during reforming and isomerization.^[^ ^76^, ^77^ ^]^ However, it offers the advantage of selectively breaking down polyolefin waste into specific short‐chain alkanes at moderate temperatures.^[^ ^78^ ^]^ The mechanisms of hydrogenolysis for polyolefins and small hydrocarbons are consistent. This process involves a sequence of elementary steps: H_2_ dissociation, alkane adsorption, and the dehydrogenation of the adsorbed alkane vis sequential C─H bond cleavage on metal surfaces. This results in quasi‐equilibrated unsaturated intermediates, which differ according to the number and positions of the removed H atoms. Both kinetic studies and simulations reveal that the C─C bond cleavage is the only kinetically relevant step.^[^ ^79^ ^]^ Flaherty and Iglesia systematically studied the hydrogenolysis of *n*‐alkanes (C_2_–C_10_) using Ir, Rh, Ru, and Pt catalysts.^[^ ^46^, ^80^ ^]^ They found that C─C bond cleavage exhibited remarkably high activation enthalpies (Δ*H* ^‡^), which slightly decreased from 257 to 214 kJ mol^−1^ as the *n*‐alkane size increased. To offset the high Δ*H* ^‡^ values, large activation entropies (Δ*S* ^‡^) are necessitated via the generation of gaseous H_2_. This results in the formation of unsaturated intermediates, which weaken C─C bonds by substituting C─H bonds with C‐metal bonds on metal surfaces.^[^ ^79^ ^]^ As the *n*‐alkane size increased from C_2_ to C_10_, the entropies varied from 118 to 673 J mol^−1^ K^−1^, with C─C cleavage rates increasing by 8 orders of magnitude.^[^ ^46^ ^]^ Importantly, hydrogenolysis rates decreased at more substituted C atoms owing to increased Δ*H* ^‡^ and Δ*S* ^‡^ values regardless of the molecule being cyclic or acyclic, following the order of ^1^C ≈ ^2^C > ^3^C > ^4^C.^[^ ^80^, ^81^, ^82^ ^]^ As a result, cleavage of terminal ^1^C─^2^C bonds is typically more favorable than that of internal C─C bonds, which leads to undesired methane formation, a phenomenon observed similarly in polyolefins. Thus, it is imperative to redirect regioselectivity away from terminal C─C bond cleavage, especially during the hydrogenolysis of highly branched polypropylene (PP).

##### Pt‐catalyzed hydrogenolysis of polyolefins

Noble metal‐based catalysts, such as Ru, Pt, and Rh, are prevalent and highly active for the hydrogenolysis of polyolefins. These monofunctional catalysts are typically dispersed on non‐acidic support materials.^[^ ^83^ ^]^ For example, Celik et al. reported that Pt nanoparticles supported on SrTiO_3_ can effectively convert both commercial high molecular weight PE and single‐use plastic bags into lubricants and waxes within 96 h at 300 °C and 11.7 bar H_2_.^[^ ^26^ ^]^ This superior performance, compared to commercially available Pt/Al_2_O_3_, was attributed to PE's stronger adsorption affinity for Pt sites on the SrTiO_3_ support. Further, the edge sites of Pt exhibited greater reactivity for PE hydrogenolysis compared to Pt facets. The cube‐on‐cube epitaxial alignment of Pt on the (100) facets of SrTiO_3_ resulted in strong interactions that effectively inhibited sintering.

**Figure 4:**
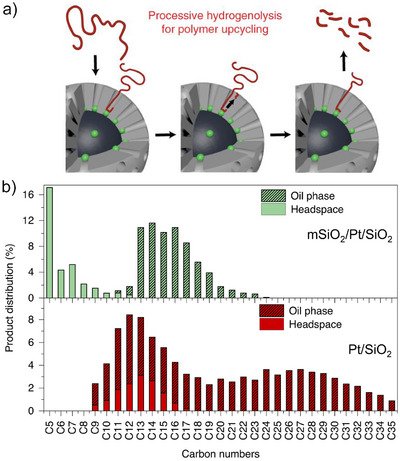
a) Processive deconstruction of polyethylene mSiO_2_/Pt/SiO_2_ catalyst. b) Combined distributions of the gas and liquid products (weighted by % yield of the products in the two phases) obtained from the hydrogenolysis of HDPE using mSiO_2_/Pt/SiO_2_ (top) and Pt/SiO_2_ (bottom) after 6 h at 250 °C and 13.8 bar H_2_. Reprinted with permission from Ref. [87]. Copyright 2020, Springer Nature.

Generally, the behavior of hydrogenolysis is influenced by the detachment of the Pt nanoparticles from the support,^[^ ^84^ ^]^ followed by their migration and subsequent sintering.^[^ ^85^ ^]^ Jaydev et al. observed that Pt supported on carbon performed better than that supported on SiO_2_ and Al_2_O_3_ for PP hydrogenolysis.^[^ ^86^ ^]^ Carbon exhibited a high capacity for hydrocarbon adsorption, while SiO_2_ and Al_2_O_3_ displayed minimal to no adsorption. As a result, Pt/C exhibited significantly enhanced selectivity toward the C_21_–C_45_ hydrocarbons. Contrary to the open environment typically seen with active metal sites on supports, Tennakoon et al. devised an ordered mesoporous shell/active site/core structure featuring Pt nanoparticles situated at the base of the mesopores (mSiO_2_/Pt/SiO_2_, Figure 4a).^[^ ^87^ ^]^ This allowed the selective hydrogenolysis of HDPE at 250 °C and 13.8 bar H_2_, resulting in a bell‐shaped distribution of liquid alkanes, primarily centered around C_12_–C_16_ (about 40% at 10% conversion). In contrast, nonporous Pt/SiO_2_ and mesoporous Pt/MCM‐41 and Pt/SBA‐15 catalysts produced a broader hydrocarbon distribution, ranging from C_18_ to C_36_ (Figure 4b). Additionally, this team synthesized three sizes of Pt nanoparticles within the mSiO_2_/Pt‐*x*/SiO_2_ matrix: small (1.7 nm), intermediate (2.9 nm), and large (5.0 nm).^[^ ^88^ ^]^ The rate of polyethylene hydrogenolysis catalyzed by the smaller Pt nanoparticles was higher than that of the larger ones, although all three catalysts exhibited similar selectivity. This indicates that the mesoscale pores within the catalytic structure affect product distribution, while the active Pt sites appear to influence the C─C bond cleavage rate. The core‐shell design improved the activity and longevity of the Pt nanoparticles, highlighting the advantages of confined environments for distinct catalyst particles in condensed‐phase reactions.

##### Ru‐catalyzed hydrogenolysis of polyolefins

In general, Ru exhibits greater activity than Pt in alkane hydrogenolysis, though it tends to catalyze substantial methane production. Ru‐based catalysts, specifically Ru/C,^[^ ^89^ ^]^ Ru/TiO_2_,^[^ ^90^, ^91^ ^]^ Ru/CeO_2_,^[^ ^92^ ^]^ and Ru/SiO_2_,^[^ ^46^ ^]^ have been shown to have high rates of alkane hydrogenolysis. Rorrer and coworkers recently reported the efficacy of Ru nanoparticles supported on carbon (5 wt% Ru/C) in the hydrogenolysis of polyethylene (average *M*_w_ ∼4000 Da) and the model compound *n*‐octadecane, operating within a temperature range of 200–250 °C under 15–30 bar H_2_.^[^ ^19^ ^]^ Reaction parameters, such as temperature, H_2_ pressure, and contact time, have been found pivotal in tailoring product distribution and selectivity. Manipulation of these reaction parameters enables both the production of liquid products and the selective hydrogenolysis to CH_4_ (at 250 °C). Overall, at intermediate temperatures low H_2_ pressures favor terminal C─C cleavage, while higher pressures lead to breaking of internal C─C bonds.

Solvents have also been shown to significantly impact hydrogenolysis kinetics and product selectivity. Jia et al. examined the hydrogenolysis of HDPE using Ru/C in various solvents. HDPE deconstruction was slow in subcritical water, attributed to its low solubility.^[^ ^21^ ^]^ Among nonpolar solvents, *n*‐hexane outperformed cyclic alkanes like methylcyclohexane and decalin for HDPE depolymerization, achieving a 90 wt% HDPE conversion to 60 wt% C_8_–C_16_ liquid hydrocarbons within 1 h at 220 °C and 30 bar H_2_. Molecular dynamics (MD) simulations indicated that PE polymers have only weak interactions with the solvent, typically in a coil. The structural resemblance between *n*‐hexane and HDPE is, therefore, speculated to uncoil significant portions of PE, enabling sufficient mobility for the polymer strands to access the Ru/C catalyst surface.

**Figure 5:**
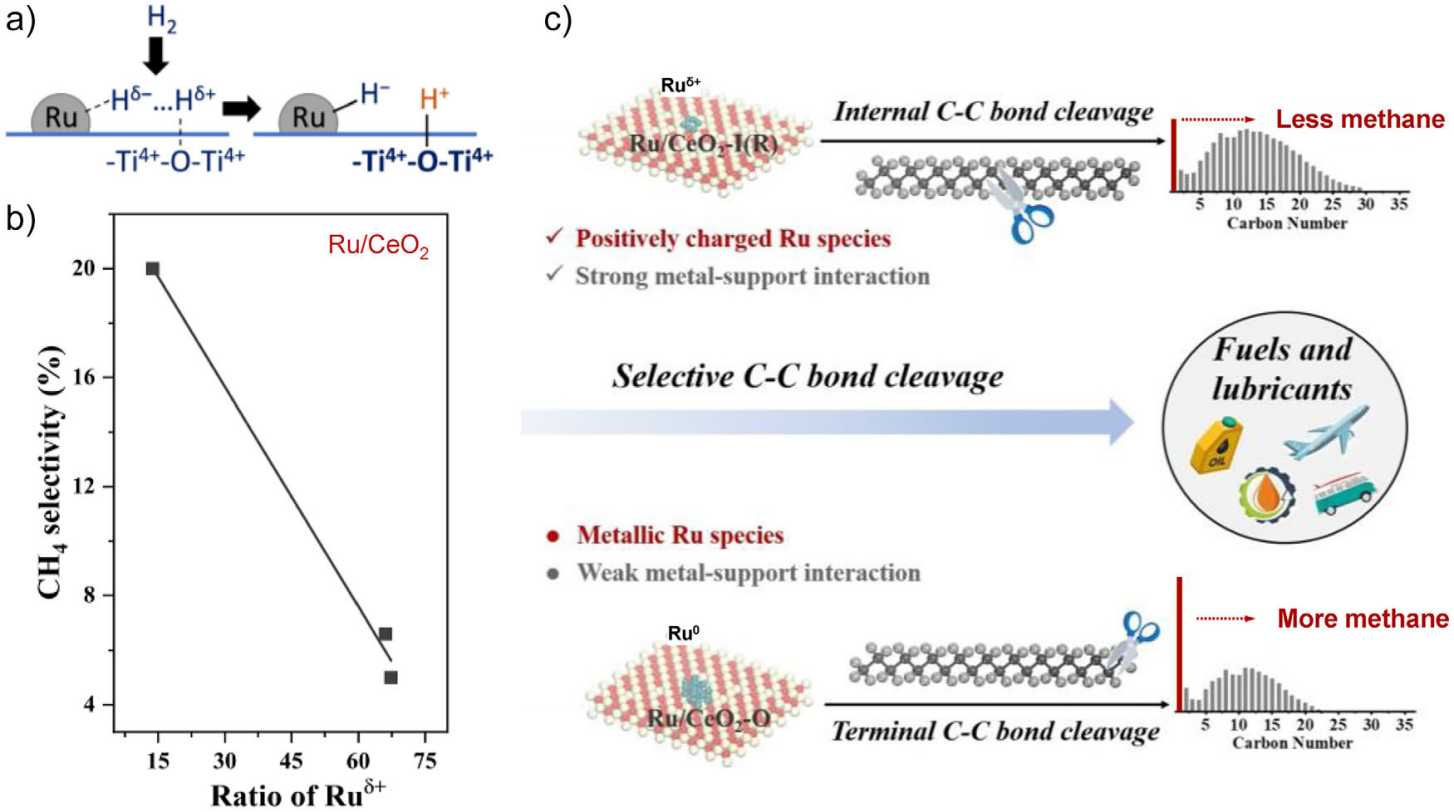
a) Hydrogen binding on the metal‐support interface of Ru/TiO_2_. Reprinted with permission from Ref. [23]. Copyright 2022, Springer Nature. b) and c) Correlation between the CH_4_ selectivity and Ru^δ+^ ratio of Ru/TiO_2_ b) and proposed reaction mechanism of PE hydrogenolysis over different Ru chemical states of Ru/CeO_2_ catalysts. Reprinted with permission from Ref. [99]. Copyright 2022, Wiley‐VCH.

The type of support critically influences the Ru activity and selectivity toward light gases and valuable liquids.^[^ ^93^ ^]^ Kots et al. found that most of the supported Ru primarily produces light C_1–4_ hydrocarbons in PP hydrogenolysis, with methane yields reaching as high as 82%.^[^ ^94^ ^]^ The methane formation decreased, however, in the order Ru/C > Ru/CeO_2_ > Ru/SiO_2_ > Ru/Al_2_O_3_ ≈ Ru/TiO_2_. Notably, Ru/TiO_2_ gave the highest liquid yield, ranging between 66% and 80% depending on the polyolefin type, and produced a total gas yield of less than 20% at 250 °C under 30 bar H_2_ pressure. The marked differences are attributed to variations in H coverage.^[^ ^23^ ^]^ H_2_ binds to the Ru/TiO_2_ metal‐support interface (Figure 5a), leading to the formation of a hydride on partially positively charged Ru (Ru^δ+^‐H^−^ ion pairs), and H^+^ on the neighboring oxygen (forming Ti–OH groups). This mechanism results in a partial reduction of TiO_2_ and a significant increase in hydrogen spillover, thereby tripling the hydrogenolysis rates. This is concluded to promote internal C─C bond cleavage and to reduce methane formation.

These results contrast with those of Nakaji et al., who reported that Ru/CeO_2_ showed much higher activity for LDPE hydrogenolysis than other Ru‐supported catalysts, such as Ru/TiO_2_, Ru/C, Ru/MgO, Ru/ZrO_2_, and Ru/SiO_2_.^[^ ^95^ ^]^ It yielded liquid fuel (C_5_–C_21_) and wax (C_22_–C_45_) at 77% and 15%, respectively, together with the low selectivity (9.8%) to gas products (C_1_–C_4_, mainly CH_4_) under a mild temperature of 200 °C and 20 bar H_2_. Chen et al. noted that the ultra‐low loading (<0.25%) of Ru/CeO_2_ resulted in decreased methane selectivity in polyolefin hydrogenolysis. In this context, Ru is characterized by its cationic nature (Ru^δ+^), inducing hydride formation.^[^ ^22^, ^96^ ^]^ Also, Ji et al. observed a maximum in the activity of CeO_2_‐supported Ru when comparing single atoms, nanoclusters, and nanoparticles in LDPE hydrogenolysis.^[^ ^97^ ^]^ Metal‐support interactions (MSI) and hydrogen spillover were identified as pivotal factors for this reaction. Specifically, MSI correlates strongly with the Ru surface states, where more electronegative Ru centers favor the activation of C─H and C─C bonds. Conversely, hydrogen spillover capability is determined by the affinity between catalysts and active H atoms. Enhancing this affinity benefits the hydrogenation of hydrocarbon surface species. While reducing Ru sizes amplifies MSI, it is speculated to reduce hydrogen spillover.^[^ ^98^ ^]^ Optimal hydrogenolysis activity is achieved when these two effects are balanced, as seen in CeO_2_‐supported Ru nanoclusters. Lu et al. further investigated the effect of the chemical state of Ru on the positions of C─C bond cleavage by comparing two Ru/CeO_2_ catalysts, each with a distinct Ru chemical state influenced by the metal‐support interaction (Figure 5b).^[^ ^99^ ^]^ Consistent with Chen^[^ ^22^ ^]^ and Vlachos’s^[^ ^23^ ^]^ findings, positively charged Ru^δ+^ species favor the hydrogenolysis of internal secondary C─C bonds and methane formation (Figure 5c). Interestingly, this is not attributed to the binding of hydrogen but to the fact that Ru^δ+^ species preferentially form bonds with the electron‐rich internal secondary carbons (a result of the electron‐donating effect from adjacent alkyl groups), rather than with the terminal carbons.

##### Earth‐abundant metal‐catalyzed hydrogenolysis of polyolefins

Earth‐abundant metals have emerged as cost‐effective catalyst alternatives to noble metals in polyethylene hydrogenolysis. While the economic advantage is evident, another significant benefit is their stability. Noble metals, despite their effectiveness, are highly susceptible to poisoning by impurities and contaminants from commercial plastic additives and waste streams. Such impurities significantly compromise the activity and selectivity of catalytic conversions.

**Zr‐based catalysts**. Dufaud et al. pioneered the demonstration that the zirconium hydride supported on silica‐alumina, [(≡SiO)_3_ZrH], as a catalyst for olefin polymerization, effectively converted LDPE and PP into alkanes via hydrogenolysis at temperatures ranging from 150 to 190 °C requiring only 1 bar H_2_.^[^ ^100^ ^]^ Specifically, the ZrH catalyst achieved a 100% conversion of LDPE (M = 125 000 Da) into saturated oligomers within 5 h or into lower alkanes over the subsequent 10 h at 150 °C. For isotactic PP (M = 250 000 Da), this catalyst achieved a 40% conversion into lower alkanes (C_1–7_) after a reaction time of 15 h at 190 °C.

**Figure 6:**
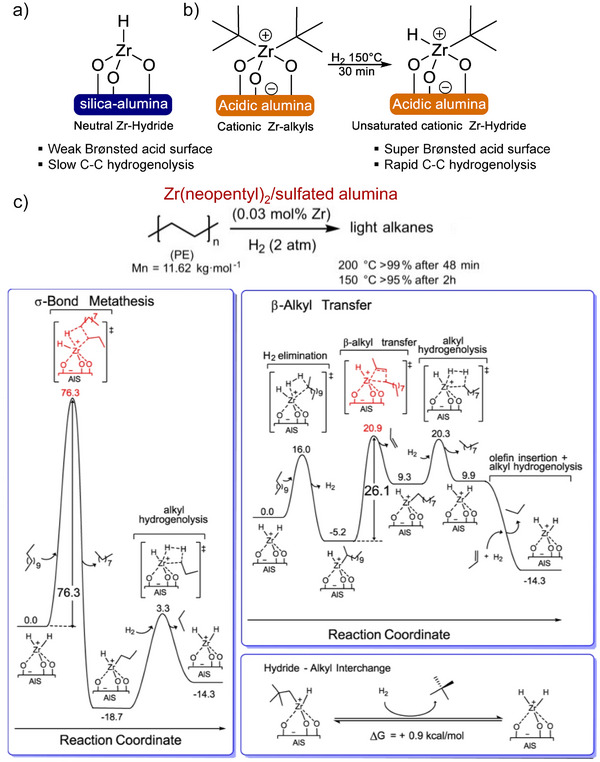
a) Zr‐hydride on a relatively weak Brønsted acidic silica‐alumina [(≡SiO)_3_ZrH]. b) The cationic Zr(neopentyl)_2_ supported on strongly Brønsted acidic sulfated alumina, pairs with its conjugate Brønsted base counteranions. This was succeeded by the generation of an electrophilic hydride via Zr–neopentyl σ‐bond hydrogenolysis. c) Calculated reaction coordinates for *n*‐dodecane hydrogenolysis catalyzed by ZrH_2_, exploring potential turnover‐limiting pathways: either C─C bond cleavage through four‐center σ‐bond metathesis or C─C scission via intramolecular β‐alkyl transfer. Additionally, the analysis includes representative computed energetics for ligand interchange between Zr alkyl and hydride. Reprinted with permission from Ref. [101]. Copyright 2022, Springer Nature.

Compared to the neutrally charged Zr attached to weak Brønsted acidic silica‐alumina, the cationic Zr alkyls on the highly Brønsted acidic sulfated alumina showed remarkable catalytic activity for polyolefins hydrogenolysis.^[^ ^101^ ^]^ For example, Zr(neopentyl)_2_/sulfated alumina produces weakly coordinating conjugate Brønsted base counteranions, thereby in situ generating sufficiently electrophilic Zr‐H species that can rapidly cleave polyolefin C─C bonds (Figure 6a,b). The catalyst successfully converted 86% of polyethylene into both liquid (43%) and volatile hydrocarbons (43%) within 50 min, under conditions of 200 °C and 2 bar H_2_ pressure. DFT analysis using *n*‐dodecane as a model revealed that the rate‐determining step in alkane/polyolefin C─C bond cleavage is governed by intramolecular ß‐alkyl transfer, instead of the σ‐bond metathesis process illustrated in Figure 6c. When considering C─C bond scission via σ‐bond metathesis (as shown in Figure 6c, left), the ZrH species confronts a notably high energy barrier of 76.3 kcal mol^−1^ (319 kJ mol^−1^). Conversely, a Zr‐sec‐dodecyl complex is efficiently formed through the H_2_ elimination step, associated with a Δ*G*° of roughly −5 kcal mol^−1^ (−21 kJ mol^−1^) and an energy barrier as minimal as 16.0 kcal mol^−1^ (67 kJ mol^−1^). Following this activation, a secondary activation within the polyolefin chain seems more plausible. The intermediate then undergoes an intramolecular β‐alkyl transfer, yielding a Zr‐alkyl and an olefin. This transition is calculated as a Δ*G*° barrier of 26.1 kcal mol^−1^ (109 kJ mol^−1^), which plays a crucial role in defining the chain deconstruction rate. Subsequently, the Zr─C bond undergoes hydrogenolysis, presenting an energy barrier of 11.0 kcal mol^−1^ (46 kJ mol^−1^). This process led to the formation of a Zr dihydride, with an associated Δ*G*° of 0.6 kcal mol^−1^ (2.5 kJ mol^−1^), producing shorter alkane chains. In summary, the hydrogenolysis process overall is exergonic, exhibiting a Δ*G*° of −14.3 kcal mol^−1^ (−60 kJ mol^−1^). While converting longer polyolefin strands into smaller alkenes, essentially reversing single‐site polymerization, is endergonic in nature, the simultaneous olefin hydrogenation ensures the entire alkane transformation, and by extension, polyethylene deconstruction, remains decidedly exergonic. Notably, DFT simulations also showed that alkene produced from ß‐alkyl transfer rapidly inserts into a Zr─H bond. This transition is barrierless and notably exergonic with Δ*G*° =  −24.2 kcal mol^−1^ (−101 kJ mol^−1^), subsequently leading to its hydrogenolysis. Experimentally, this agrees with observations that alkenes are never detected throughout the reaction.

**Co‐based catalysts**. Zichittella et al. investigated several bulk metal oxides for their effectiveness in catalytic *n*‐C_24_ hydrogenolysis.^[^ ^25^ ^]^ They found that cobalt oxide (Co_3_O_4_) was active but predominantly produced methane at 250 °C and 40 bar H_2_. This suggested that Co_3_O_4_ primarily favors a terminal C─C bond cleavage. While dispersed cobalt oxide on a redox‐inert support allows for a range of oligomeric products. Borkar et al. found that silica‐supported cobalt (5 wt% Co/SiO_2_) led to 55% liquid products (C‐mol basis) and restricted gas yields of ∼19% under conditions of 275 °C, 30 bar H_2_ and 8 h. Borkar et al., comparing Co_3_O_4_ with Co/SiO_2_, note that there was a marked shift in selectivity from primarily gaseous to predominantly liquid products under identical reaction conditions, showing 7% versus 53% liquid‐phase selectivity.^[^ ^102^ ^]^ The polymer chain was hypothesized to adsorb weakly on the redox‐inert SiO_2_ support. This facilitates desorption of the severed branches, avoiding successive terminal cleavage and resulting in liquid products. Although Co/SiO_2_ transitioned from Co_3_O_4_ to CoO during the reaction, this change in valence state had only a minor impact on the hydrogenolysis activity.

**Figure 7:**
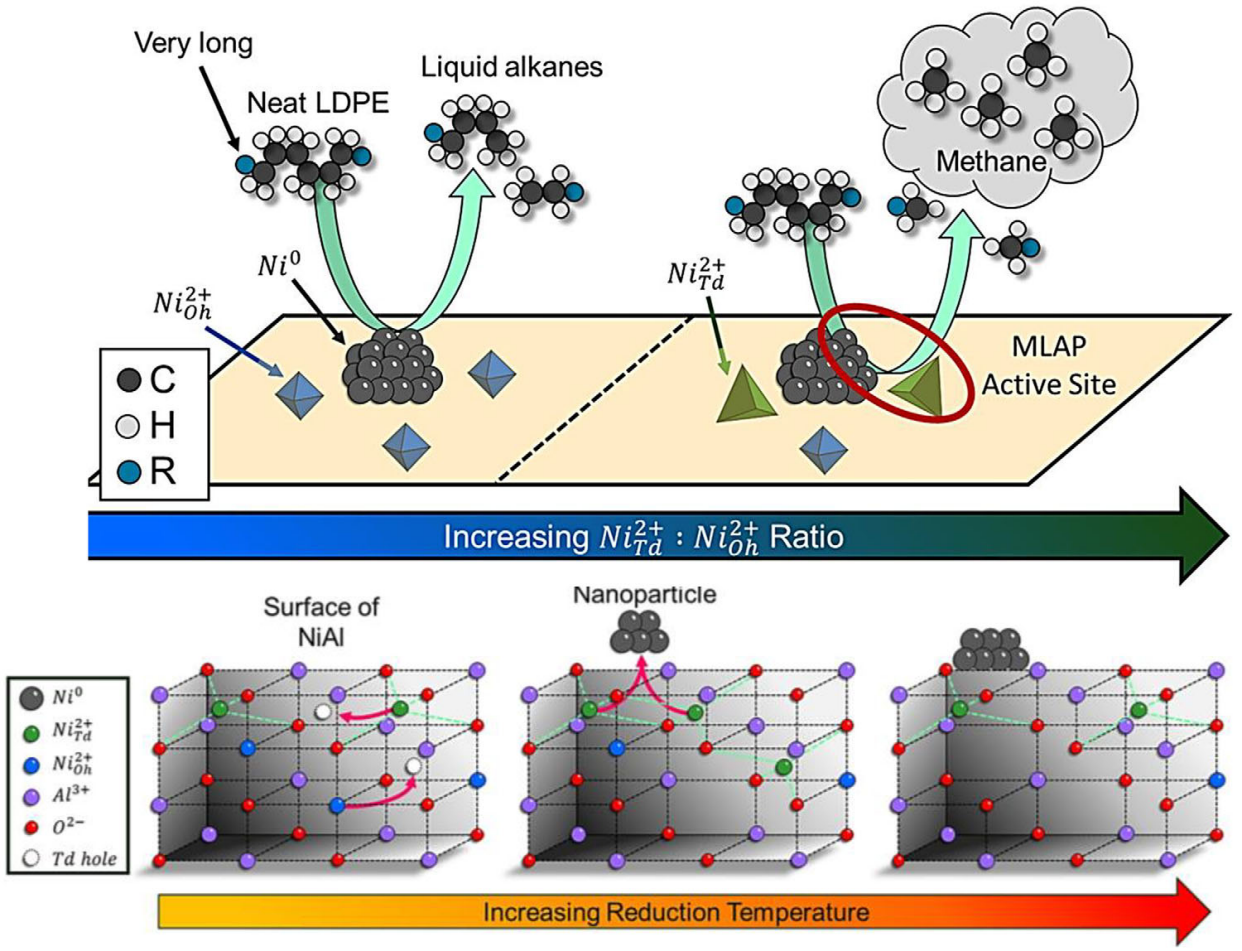
The proposed mechanism of metallic Ni° favors the hydrogenolysis of internal C─C bonds while Ni^2+^ drives the hydrogenolysis of terminal C─C bonds, leading to methane production (top). Conceptual depiction of structural transformations and Ni nanoparticle generation in near‐surface layers (bottom). Note that Ni_Td_^2+^‐nickel cations in tetrahedral centers [NiO_4_] weakly interact with nearby Al. Ni_Oh_^2+^‐nickel cations in [NiO_6_] octahedral centers that strongly interact with nearby Al. Reprinted with permission from Ref. [104]. Copyright 2023, American Chemical Society.

**Ni‐based catalysts**. Vance et al. showed that Ni/SiO_2_ had catalytic activity hydrogenolysis of LDPE comparable to that of supported noble metals.^[^ ^103^ ^]^ Specifically, a sample tagged 15Ni/SiO_2_ achieved high yields of diesel (C_9_–C_22_) and lubricant (C_20+_) range hydrocarbons with maximum yields of 65 wt% and low methane yields of 17% after 9 h at 300 °C, 30 bar H_2_. Later, his team reported a nickel aluminate catalyst, wherein Ni atoms were closely associated with Al atoms, effectively suppressing methane formation, leading to less than 5% methane selectivity during LDPE hydrogenolysis under consistent conditions.^[^ ^104^ ^]^ Lowering the ex situ reduction temperature from 550 to 350 °C significantly decreased methane selectivity, from ∼33% to less than 5%, while leaving the yields of liquid alkanes (C_6_–C_35_) unchanged. Ni nanoparticles form through the migration of cationic Ni within the topmost nanometers (Figure 7). Initially, this cationic nickel predominantly exists as octahedral Ni^2+^ (denoted as Ni_Oh_^2+^) in the subsurface, and these species are inactive for polyethylene hydrogenolysis. As the reduction temperature increases, the amount of tetrahedral Ni^2+^ (denoted as Ni_Td_^2+^) increases, and the higher concentration results in the formation of metallic Ni nanoparticles (Ni^0^) upon reduction with H_2_. Higher reduction temperatures tend to produce more Ni^0^ and alter the fractions of the Ni_Oh_^2+^ and Ni_Td_^2+^ in the subsurface. Interestingly, a strong linear correlation exists between the Ni_Td_^2+^/Ni_Oh_^2+^ ratio and CH_4_ selectivity, with higher fractions of Ni_Td_^2+^ resulting in increased CH_4_ selectivity. These findings indicate that metallic Ni^0^ appears to be responsible for hydrogenolysis, while the Lewis acid sites associated with surface Ni_Td_^2+^ are located near the periphery of Ni^0^, generating metal‐Lewis acid pairs (MLAPs) that favor terminal C─C scissions, leading to excessive CH_4_ generation. This highlights the pivotal role of catalytic support and site pairing in determining both activity and selectivity.

#### 3.2.2 Hydrocracking

**Figure 8:**
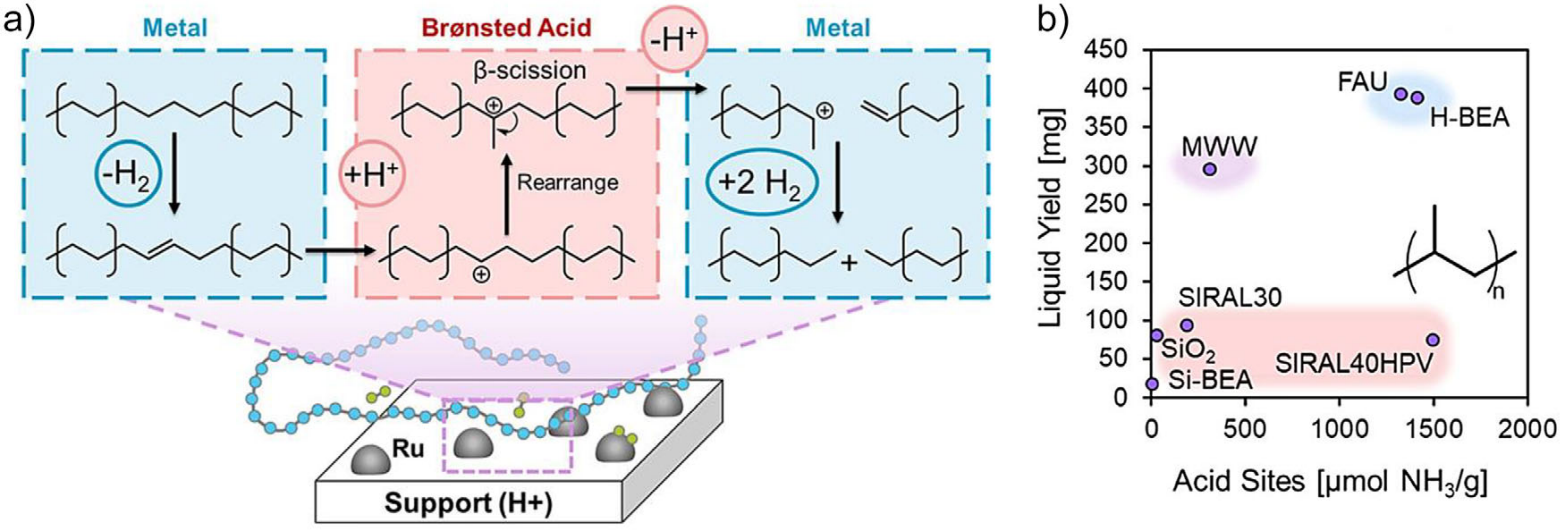
a) Simplified schematic of hydrocracking over metal nanoparticles (e.g., Ru) supported on Brønsted acidic supports. b) Product yields for the reaction of PE (avg. *M*_w_ 4000 Da) over 5 wt% Ru on supports of varying acidity and structure. Reaction conditions: 200 °C, 16 h, 30 bar H_2_, 700 mg PE, 50 mg catalyst (50 mg Ru/SiO_2_, 50 mg FAU for physical mixture). Reprinted with permission from Ref. [28]. Copyright 2022, American Chemical Society.

Although the hydrogenolysis strategy is widely used for polyolefin deconstruction, it lacks isomerization and thus tends to form unbranched alkanes. As highlighted above, kinetic coupling of Brønsted acid‐catalyzed C─C bond cleavage with hydrogenation on the metal function provides an efficient method to offset the endothermicity of the cleavage process,^[^ ^105^ ^]^ enhancing selectivity for nonterminal C─C bond cleavage via β‐scission pathways and consequently minimizing methanation. Hydrocracking catalysts typically feature metal sites supported on solid acids, such as sulfated metal oxides, mixed oxides, or crystalline zeolites.^[^ ^106^, ^107^, ^108^, ^109^ ^]^ Their functionality necessitates the cooperation between metal and acid sites for both C─C bond cleavage and the processes of hydrogen addition and abstraction in reactants and products (Figure 8a).^[^ ^28^ ^]^ Product distribution is determined by the rates of C─C bond cleavage, isomerization, and hydrogenation/dehydrogenation activities. These rates, in turn, are influenced by the nature of the acid and metal sites encompassing their acid strengths and concentrations and the catalytic behavior of metals in hydrogenation and dehydrogenation.^[^ ^110^ ^]^ These factors are further modulated by the reaction temperature, pressure, and the chemical potentials of reactants and products.

##### Ru‐catalyzed hydrocracking of polyolefins

In addition to its function as a hydrogenolysis catalyst, Ru has been extensively studied for its role in polyolefin hydrocracking. Rorrer et al. investigated Ru catalysts supported on a series of supports known for their strong Brønsted acidity.^[^ ^28^ ^]^ Specifically, Ru supported on Brønsted acidic FAU and H‐BEA resulted in yields of liquid alkanes at 67% and 51%, respectively. In comparison, using an inert silica support (Ru/SiO_2_) yielded only 33% under identical conditions of 200 °C, 30 bar H_2_, and 16 h for PE conversion. For reactions involving PP hydrocracking, a distinct and positive correlation is evident between the liquid yield and the concentration of acid sites, as illustrated in Figure 8b. This observation agrees with the mechanism that Brønsted acid sites catalyze cracking via carbenium ions, suppressing methane formation and favoring central C─C bond cleavage, leading to increased production of liquid‐range alkanes.

**Figure 9:**
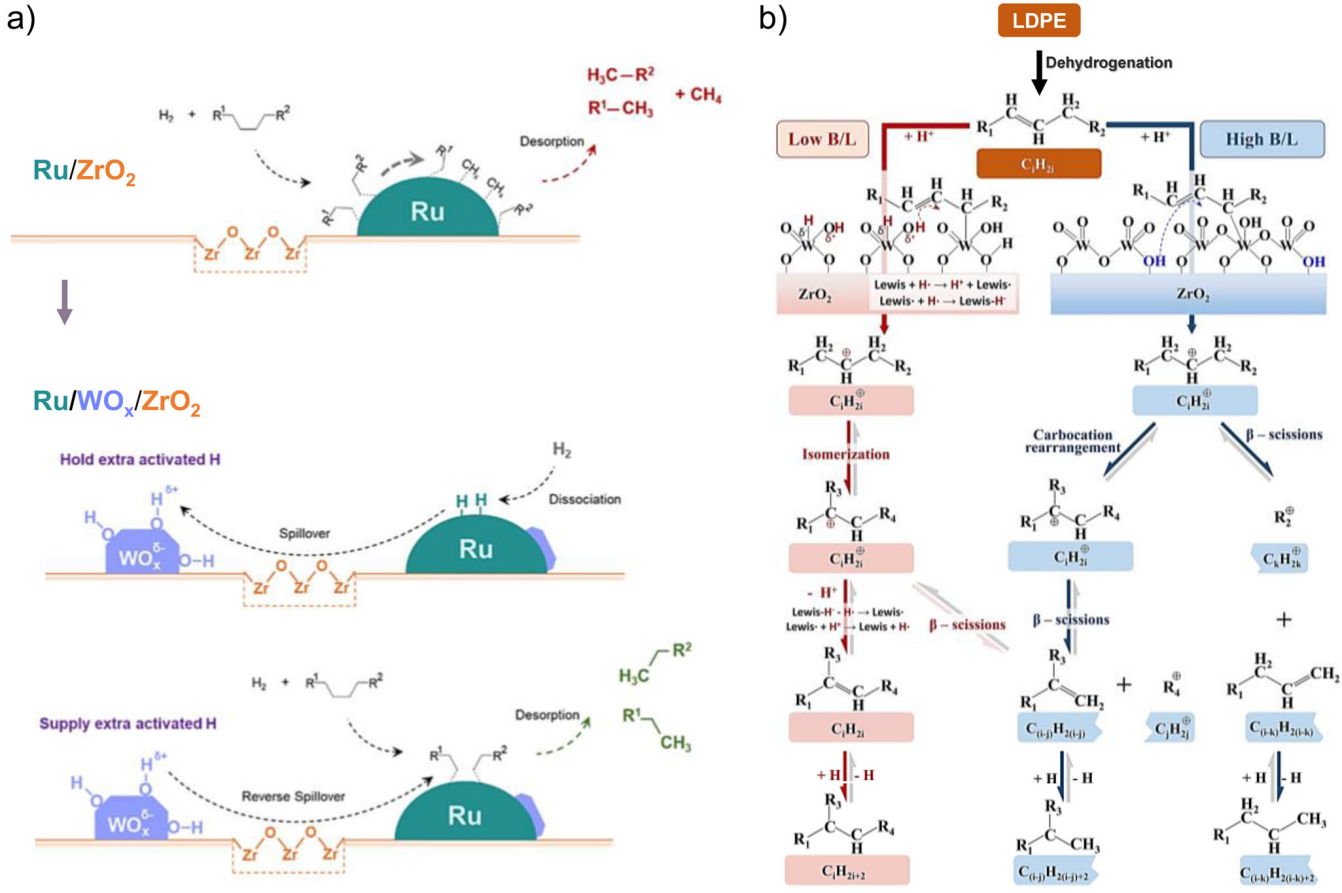
a) Comparison of proposed reaction mechanism for LDPE hydrocracking on Ru supported by zirconia (ZrO_2_) and tungstated zirconia (WO*_x_*‐ZrO_2_). Reprinted with permission from Ref. [20]. Copyright 2021, American Chemical Society. b) The Brønsted/Lewis acid sites (B/L) ratio of WO*_x_*‐ZrO_2_ governs reaction pathways and product distributions. Reprinted with permission from Ref. [111]. Copyright 2024, Elsevier.

Wang et al. reported that Ru‐supported zirconia catalysts doped with Ti, Nb, Ce, W, V, Mo, and Fe for polyolefin deconstruction.^[^ ^24^ ^]^ In particular, tungstated zirconia (Ru/WO_3_/ZrO_2_) significantly minimizes methane production and yields branched liquid wax/lubricant hydrocarbons in the hydrogenolysis of LDPE. This performance outperformed Ru supported on bare zirconia, tungstated silica, HY zeolite, and mesoporous Al‐MCM‐41, suggesting that acidity is not the primary factor in methane suppression.^[^ ^20^ ^]^ They proposed that while WO*_x_* does not promote Ru‐catalyzed C─C bond activation (which is rate‐limiting under high pressures), its high dispersion increases the H storage capacity in surface hydroxyls, a consequence of hydrogen spillover from Ru to WO*_x_* (Figure 9a). Specifically, when Ru and WO*_x_* species are highly dispersed on ZrO_2_, the partially reduced ZrO_2_ sites mediate the transfer of H^δ+^ species and electrons between Ru and WO*_x_*. These H^δ+^ species can reversibly spillover between Ru and WO*_x_* clusters, facilitating efficient hydrogenation and product desorption. This prevents Ru from generating olefins susceptible to hydrocracking and isomerization on the Brønsted acid sites. Shang et al. highlighted the critical role of the local acid site environment in governing reaction pathways and product distributions (Figure 9b).^[^ ^111^ ^]^ They observed that variables such as calcination temperature and WO*_x_* loading influence WO*_x_* surface density and the Brønsted to Lewis acid site ratio (B/L ratio). A higher B/L ratio promotes LDPE hydrocracking through synergistic acid site interactions. Conversely, excessive WO*_x_* leads to WO_3_ crystallization and lowers the B/L ratio, favoring hydrogen transfer over β‐scission, thereby hindering LDPE hydrocracking. Overall, acid sites are essential to facilitate polymer isomerization and catalyze chain truncation via β‐scission of carbenium ions, producing unsaturated alkenes, which are subsequently hydrogenated at metal sites.^[^ ^75^ ^]^

##### Pt‐catalyzed hydrocracking of polyolefins

**Figure 10:**
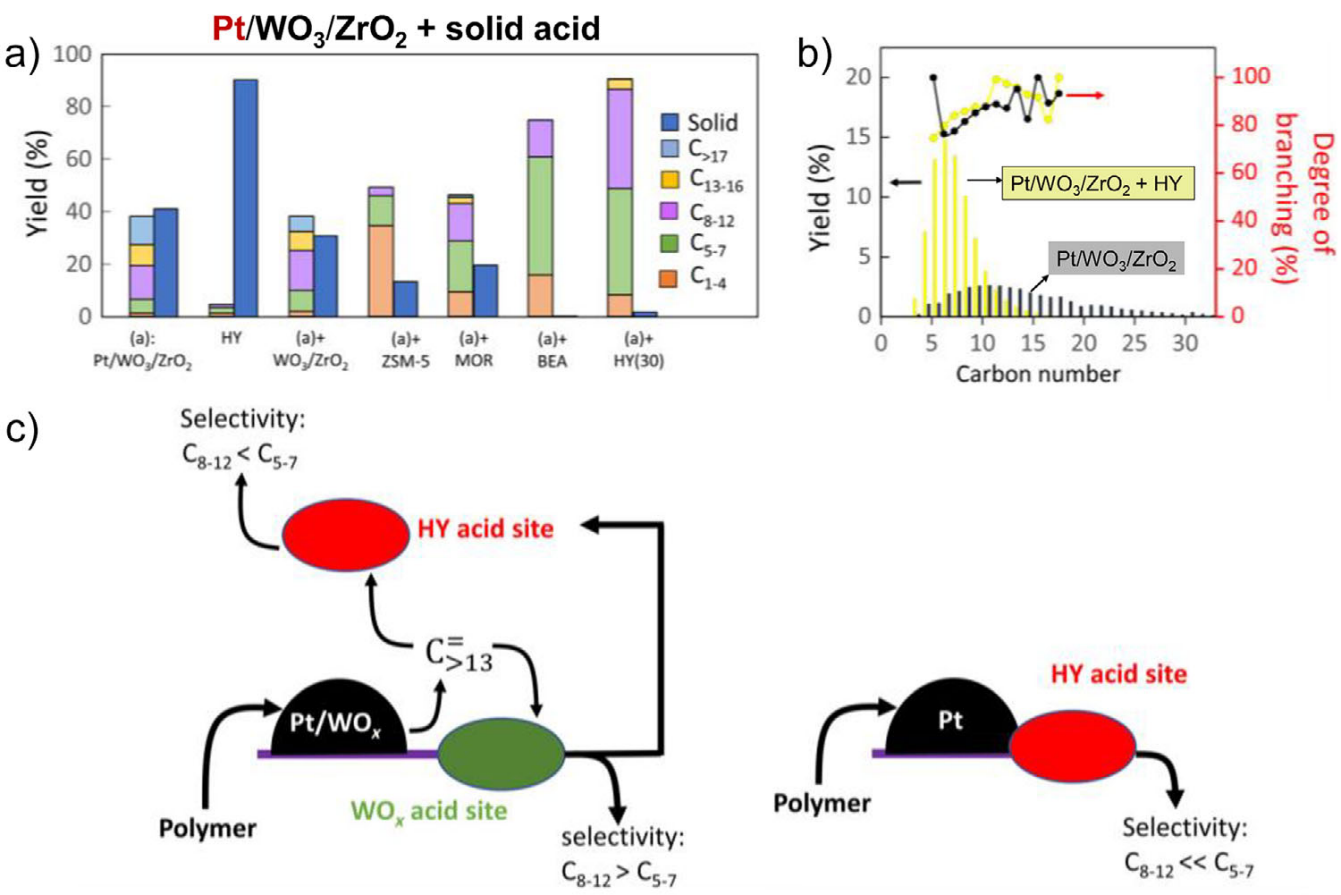
Hydrocracking of LDPE over Pt/WO_3_/ZrO_2_ mixed with various solid acid catalysts including HY, HBEA, H‐MOR, HZSM‐5, and WO_3_/ZrO_2_. a) Reaction conditions: 250 °C, 30 bar H_2_, 2.0 g LDPE, 0.1 g Pt/WO_3_/ZrO_2_, 0.1 g solid acid, and reaction time of 2 h. b) Product yields and degree of isomerization by carbon number for pure Pt/WO_3_/ZrO_2_ (black) and Pt/WO_3_/ZrO_2_ mixed with HY(30) in a 1:1 mass ratio (yellow). c) Depiction of main intermediates diffusing over Pt/WO_3_/ZrO_2_ + HY(30), compared with the absence of WO_3_ that intimate contact between Pt particles and HY. Reprinted with permission from Ref. [27]. Copyright 2021, AAAS.

Nevertheless, Ru supported on Brønsted acidic supports shows limited isomerization activity compared to its Pt analogous.^[^ ^73^ ^]^ Ru is characterized by pronounced metal−carbon bond strength leading to deep dehydrogenation and subsequent C─C scission, thereby inhibiting its production of olefin intermediates to hydrocracking and isomerization at Brønsted acid sites.^[^ ^112^ ^]^ Zhou et al. found that PE chains bind to oxygen vacancies in Pt/WO_3_, leading to the formation of C═O and subsequently C═C intermediates, which are then converted to alkanes via hydrogenation. This process achieved complete HDPE conversion, producing 88.9% fuel at 200 °C under 30 bar H_2_ for 20 h.^[^ ^29^ ^]^ Vlachos et al. identified Pt/WO_3_/ZrO_2_ as a bifunctional catalyst for LDPE hydrocracking, producing branched alkanes suitable for fuels and lubricants.^[^ ^113^, ^114^ ^]^ Mechanical mixing Pt/WO_3_/ZrO_2_ with HY zeolite significantly improved catalytic activity, reducing solid residue and shifting product distribution toward gasoline‐range molecules (Figure 10).^[^ ^27^ ^]^ At these conditions, it was found that HY zeolite alone had lower activity (91% solid residue), indicating a synergy between Pt/WO_3_/ZrO_2_ and HY zeolite. Within the zeolite pores, rapid cracking into smaller C_5_–C_7_ alkenes occurs, while the slower cracking rate on Pt/WO_3_/ZrO_2_ is attributed to weaker acid sites and the lack of activity enhancement by the micropore solvation. The efficiency of LDPE conversion also varies with zeolite pore size (HY ≈ HBEA > H‐MOR > HZSM‐5). The proposed mechanism suggests that LDPE initially cracks on Pt/WO_3_/ZrO_2_ and then diffuses into the zeolite pores. Similar to the Pt/WO_3_/ZrO_2_ + HY observations, initial cracking is catalyzed on external acid sites before diffusion of smaller product molecules into the microporous protons occurs. Indeed, directly supported Pt on HY induces diffusion limits for larger hydrocarbons, favoring lighter alkane production.^[^ ^115^ ^]^ Wu et al. demonstrated that introducing Ce into Pt/HY catalysts markedly enhanced adsorption of intermediates, achieving 81% selectivity toward liquid fuels from fully converted LDPE at 300 °C in 2 h. This performance significantly surpasses that of Pt/HY alone under identical conditions, which results in only a 40% conversion of LDPE with a 21% selectivity for liquid fuels.^[^ ^116^ ^]^ The communication via diffusion between the metal and acid sites is pivotal for effective hydrocracking processes, particularly with bulky polymer molecules.

##### Noble metal‐free catalysts for polyolefins hydrocracking

Noble metal‐free catalysts offer a cost‐effective alternative for polyolefin hydrocracking; however, they are rarely studied in the context of polyolefin hydrocracking. This limited attention stems from their slower dehydrogenation and hydrogenation kinetics compared to noble metal‐based catalysts, resulting in reduced hydrocracking efficiency. Recent studies have primarily focused on metal‐free zeolites,^[^ ^117^, ^118^, ^119^ ^]^ supported nickel and cobalt,^[^ ^111^, ^120^, ^121^ ^]^ and molybdenum sulfides.^[^ ^122^ ^]^

Duan and his coworkers developed ZSM‐5 zeolite nanosheets for PE cracking into olefins at 280 °C under a flowing hydrogen carrier gas.^[^ ^117^ ^]^ More specifically, the Brønsted‐acidic ZSM‐5 nanosheets, characterized by a high external surface area and abundant micropores, catalyzed PE cracking on the zeolite surface. This process facilitated the formation of intermediates that diffused into the micropores, where they underwent further cracking into small molecules. By promoting rapid transport and conversion within the micropores, the ZSM‐5 nanosheets effectively suppressed intermediate accumulation on the surface, thereby minimizing coke formation. The study also revealed that reactions conducted in the absence of H₂ showed significantly lower yields of C_1_–C_7_ hydrocarbons compared to those performed under H₂, while producing substantial wax fractions. Based on these observations, the authors proposed that H₂ participates in the cracking mechanism by suppressing the formation of polycyclic aromatic intermediates within the zeolite micropores.

Lee's team found that HZSM‐5 achieved only 14% conversion of *n*‐hexadecane (*n*‐C_16_) as a model substrate at 275 °C and 4.5 MPa H_2_ pressure, while the conversion increased to 98% when the temperature was raised to 375 °C.^[^ ^121^ ^]^ Similar to the findings of Duan et al., the conversion of *n*‐C_16_ was slightly reduced from 98% to 93% when conducted under N_2_ compared to the reaction conducted under H_2_. They also found that Co and Ni nanoparticles supported on amorphous silica‐alumina, HY, and HZSM‐5 were less active than the zeolite supports alone. However, these nanoparticles significantly increased the proportions of saturated hydrocarbons in the final product distribution. These findings further demonstrate that Co and Ni nanoparticles are unable to efficiently cleave C─C bonds but can effectively catalyze the hydrogenation of unsaturated bonds formed during the cracking process.

Interestingly, Tan's group found that at a lower temperature (200 °C) and H_2_ pressure (10 bar), the zeolite (HFAU, HZSM‐5) could catalyze cracking of LDPE without metal to form predominately C_3_–C_7_ gaseous products.^[^ ^118^ ^]^ Similar carbon selectivities were also observed under N_2_, though increased selectivity to alkenes at a given carbon number was observed. Regardless, there was significant alkane production in metal‐free zeolite reactions (under either H_2_ or N_2_), indicating hydrogenation and/or hydride transfer events by acid sites.^[^ ^119^ ^]^ Through external site titrations, they demonstrated that initial activation of polymer species occurs on external acid sites before molecules diffuse into micropores for additional cleavage events, which can be facilitated with incorporation of mesopores. Cen's team showed that PE can be converted into gasoline‐range alkanes with yields of up to 81% and a selectivity of 99% at 240 °C (without the presence of H_2_) by using a layered self‐pillared zeolite (LSP‐Z100). In this process, the polymer itself serves as the hydrogen source.^[^ ^123^ ^]^ They reasoned that the PE is initially activated by the open framework tri‐coordinated aluminum sites of zeolites, followed by β‐scission and isomerization over Brønsted acid sites, and then undergoes hydrogen transfer via a self‐supplied hydrogen pathway.^[^ ^124^, ^125^ ^]^

It is evident that the C─C cleavage step should proceed through a Brønsted acid‐catalyzed cracking mechanism rather than hydrogenolysis in zeolite catalysis; H_2_ is only required for the hydrogenation of cracked unsaturated hydrocarbons (or hydride transfer to carbenium ions) and does not directly participate in the C─C bond cleavage. Given that the cracking of PE is a thermodynamically unfavorable process, it is plausible that the unfavorable thermodynamics at low temperatures may be largely compensated by hydrogenation and hydrogenolysis at such low temperatures. Therefore, it is essential to verify the presence of metal impurities in the zeolite catalysts, the polyolefins, and the reactor throughout the entire process.

Despite advancements in hydrocracking, a key challenge remains in balancing and controlling the strength and concentration of acid and metal sites to enable lower reaction temperatures and H_2_ pressures. Addressing this challenge requires a deep understanding of the interplay between these sites and the integration of acid‐catalyzed C─C bond modifications with metal‐catalyzed hydrogen addition. Variations can be introduced by combining active sites on solid acid surfaces, or in hierarchical zeolite mesopores, and through solvent interactions. The relative rates of catalytic functions and their interactions raise essential questions rooted in reaction‐diffusion theories.^[^ ^119^ ^]^ Utilizing model hierarchical or nano‐sized zeolites with model compounds enables fine‐tuning of functional separation, strength, and concentration.

### 3.3 Cyclization/Aromatization

**Figure 11:**
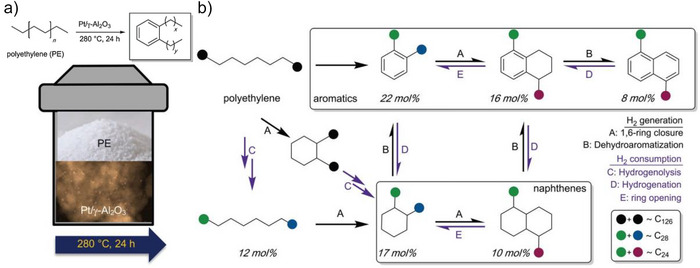
a) Tandem hydrogenolysis/aromatization for converting PE to long‐chain alkyl aromatics under solvent‐free and H_2_‐free conditions. Note that the schematic illustrates the reactor and product fractions. This includes photographs of the powdered polymer and liquid products, along with a transmission electron micrograph of the catalyst. b) Proposed pathway of polyethylene to alkylaromatics and alkylnaphthenes through tandem hydrogenolysis/aromatization via dehydrocyclization. Reprinted with permission from Ref. [30]. Copyright 2020, AAAS.

The strategy of using metal‐catalyzed C─C cleavage in polyolefins followed by cyclization and intermolecular rearrangements offers a promising approach for producing high‐value aromatics. Zhang et al. reported this tandem hydrogenolysis/aromatization method enables thermodynamic and kinetic coupling through hydrogen redistribution at relatively low temperatures (280 °C) using a bifunctional Pt/γ‐alumina catalyst (Pt/γAl_2_O_3_, Figure 11a).^[^ ^30^ ^]^ This bypasses the reliance on H_2_, which has been a significant obstacle for hydrogenolysis technologies.^[^ ^126^ ^]^ Specifically, after 24 h at 280 °C, the tandem catalytic system produced liquid yields of 80, 69, and 55 wt% for low‐molecular‐weight PE, LDPE, and HDPE, respectively. These yields had an average carbon number of approximately C_30_, with alkyl aromatic selectivities of approximately 57, 44, and 50 mol%, respectively.

The dehydrogenation of PE predominantly produces either an olefin, though its formation at adjacent polymer chain sites was not observed, or a cycloalkane due to dehydrogenation at non‐adjacent sites. Subsequently, the olefin formed may undergo rapid cyclization, potentially aided by the acid sites on the support. This process releases H_2_, which participates in the exothermic C─C bond hydrogenolysis, truncating the polymer chain. When a cyclopentane ring forms, it likely converts into cyclohexane via acid catalysis, eventually forming dialkylaromatics (Figure 11b).^[^ ^127^ ^]^ The equilibrium between the exothermic and endothermic reactions allows the overall PE conversion for polyethylene.

Direct conversion of PE to aromatics appears to require elevated temperatures since *n*‐alkane aromatization is an endothermic process.^[^ ^128^ ^]^ Thermodynamic values, calculated for the conversion of linear PE chains to alkyl aromatics at 280 °C under 1 bar H_2_, are Δ*H*° = 246 kJ mol^−1^ and Δ*G*° = 31 kJ mol^−1^.^[^ ^30^ ^]^ It has been suggested that the reaction occurs in tandem with the hydrogenation by H_2_, sourced from the PE chains that act as an internal hydrogen reservoir. Despite the dehydrogenation of PE to produce H_2_ endothermically at moderate temperatures, it can proceed to an extent when facilitated by small Pt nanoparticles (approximately 1 nm in diameter). In the presence of H_2_, the thermodynamic values for C─C bond hydrogenolysis are estimated to be Δ*H*° = −49 kJ mol^−1^ and Δ*G*° = −74 kJ mol^−1^. As a result, at 280 °C, aromatization becomes favorable (Δ*G*° = 0) if even 10% of the generated H_2_ is used in PE hydrogenolysis. Product analysis indicates that over 90% of the produced H_2_ was used in PE deconstruction through hydrogenolysis, making the tandem process thermodynamically favorable.

**Figure 12:**
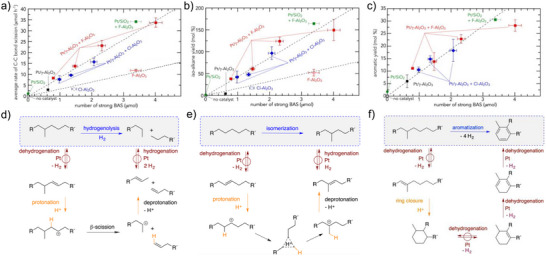
a–c) The number of strong Brønsted acid sites controls the kinetics of triacontane (*n*‐C_30_H_62_) conversion and e–f) Proposed mechanisms for C─C bond scission via hydrogenolysis, isomerization and aromatization. a) The average rate of C─C bond scission and d) the proposed pathway for C─C bond scission; b) the *iso*‐alkane yield and e) proposed pathway for alkane isomerization; c) the aromatic yield and f) proposed pathway for aromatization. Reprinted with permission from Ref. [11]. Copyright 2023, Elsevier.

To clarify the role of acid sites in the tandem mechanism and the synergy between the strength and proximity of acid and metal sites, further investigations were conducted using a series of acidic supports for Pt nanoparticles.^[^ ^11^ ^]^ Halogenated γ‐Al_2_O_3_, modified with Cl or F, showed enhanced surface acidity, in close similarity to the classic petroleum reforming. This resulted in markedly higher average rates of C─C bond cleavage, skeletal isomerization, and alkyl aromatic production. The rates of each type of reaction show a limited correlation with the number of Lewis acid sites (LAS) or weak to moderate Brønsted acid sites (BAS). However, they exhibit an approximately linear relationship with the presence of strong BAS, which is modulated by the physical mixing of Pt/γ‐Al_2_O_3_ with halogenated alumina (Figure 12). The proposed pathways suggest that Pt sites primarily catalyze hydrogenation/dehydrogenation, while strong BAS facilitates the protonation of alkenes, forming carbenium ions that undergo C─C bond cleavage, isomerization, and cyclization.^[^ ^129^ ^]^ Chang and Rangarajan employed machine learning‐based thermochemistry calculations to investigate potential reaction pathways for the dehydroaromatization of *n*‐decane, a model polyolefin, into aromatics.^[^ ^130^ ^]^ From the 24000 reactions and 3759 species generated, they identified 78 aromatic molecules that undergo a sequence of dehydrogenation, β‐scission, and cyclization. Specifically, thermodynamically, decane predominantly follows this pathway (Figure 14): (i) two dehydrogenation steps, (ii) a β‐scission yielding an ethylene molecule, (iii) a third dehydrogenation, (iv) another β‐scission producing an ethylene molecule, (v) cyclization, and (vi) a final dehydrogenation resulting in benzene. Among them, the most thermodynamically challenging step was found to be either the first or second dehydrogenation, given that its Δ*H*_rxn_ was higher than that of the other reaction steps.

The bifunctional nature of the metal‐acidic catalyst is crucial for the tandem hydrogenolysis/aromatization process. Catalysts with high acid site concentrations, like Pt/HZSM‐5 and Ni/Cu/HBETA, predominantly facilitate cracking toward BTX (benzene, toluene, and xylene) but also result in a significant yield of light gases.^[^ ^32^, ^131^ ^]^ Catalysts like Pt/SiO_2_ or Pt/C, which lack acid sites, fail to effectively deconstruct polyethylene, even when the size of the Pt nanoparticles is comparable.

**Figure 13:**
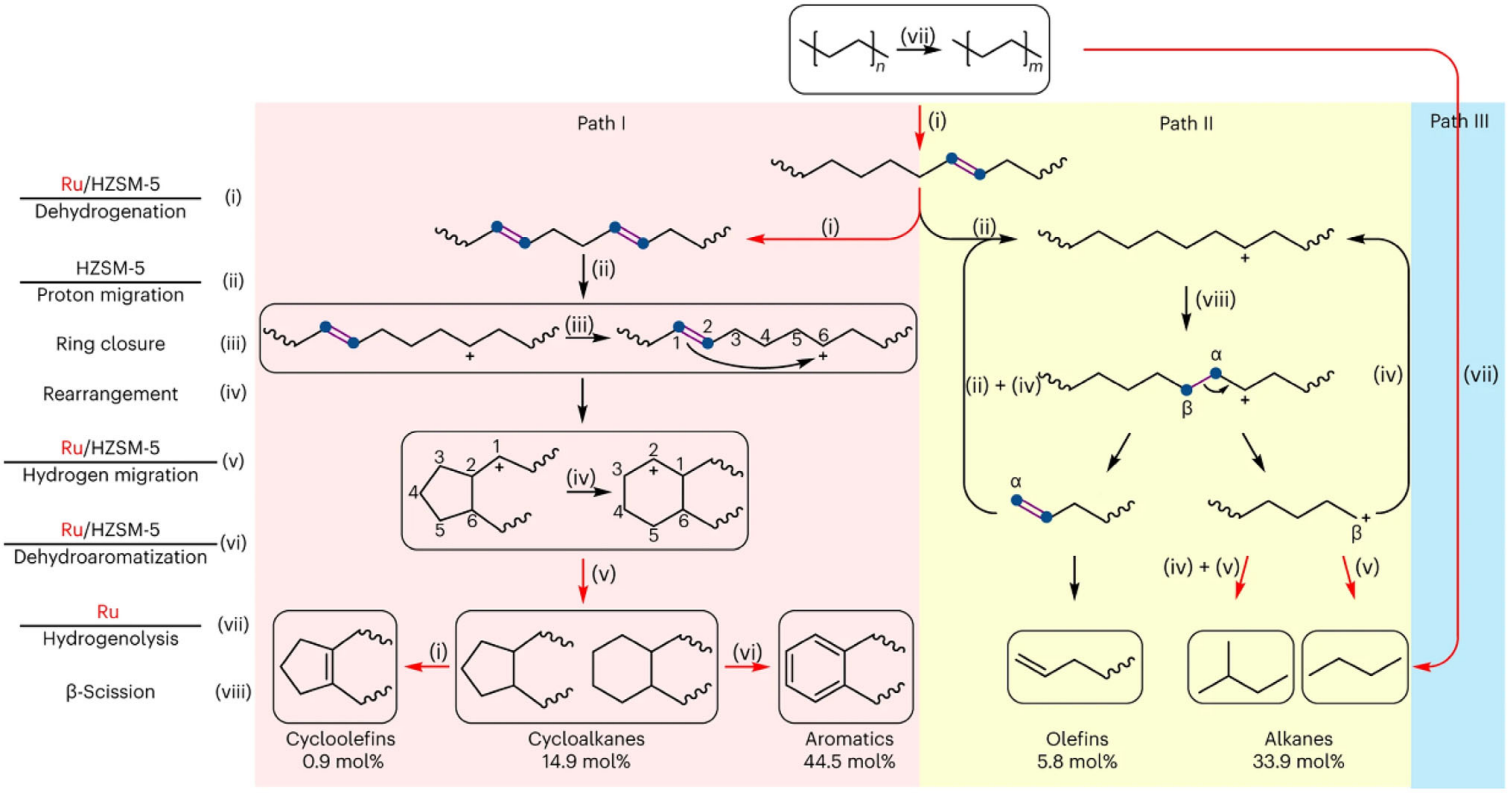
Reaction pathways for HDPE upcycling over Ru/HZSM‐5. The red arrows represent the pathways enhanced by Ru. Paths I, II, and III illustrate the cyclization, β‐scission, and hydrogenolysis reactions, respectively. Steps (i)–(viii) are the elementary reactions, namely dehydrogenation, proton migration, ring closure, carbenium ion rearrangement, hydrogen migration, dehydroaromatization, hydrogenolysis, and β‐scission. To the left of the figure, the species above the line shows the active component responsible for the elementary step shown below the corresponding line. The steps without a line are spontaneous, not requiring catalysts. The selectivity for different products was determined over Ru/HZSM‐5(300) in HDPE upcycling at 280 °C for 24 h. Reprinted with permission from Ref. [31]. Copyright 2023, Springer Nature.

Du et al. demonstrated that Ru nanoparticles supported on low‐alumina HZSM‐5 can efficiently catalyze the solvent‐ and hydrogen‐free upcycling of HDPE into linear (C_1_–C_6_) and cyclic (C_7_–C_15_) hydrocarbons at 280 °C for 24 h. This process achieved up to 60.3 mol% selectivity for monocyclic hydrocarbons, which included cycloalkanes (14.9 mol%), cycloolefins (0.9 mol%), and aromatics (44.5 mol%).^[^ ^31^ ^]^ Remarkably, Ru/HZSM‐5 achieved a selectivity of up to 60.3 mol% for valuable monocyclic hydrocarbons, including cycloalkanes (14.9 mol%), cycloolefins (0.9 mol%), and aromatics (44.5 mol%). In contrast, ZSM‐5 alone gave a selectivity of 42.5 mol%, with aromatics accounting for only 24.0 mol%. Mechanistic studies show that Ru sites promote the dehydrogenation of HDPE to produce C═C bonds, while the Brønsted acid sites on HZSM‐5 are key to transforming these bonds into carbenium ions (Figure 13). If a carbenium ion is positioned at an appropriate distance (2–4 carbon atoms) from a neighboring C═C bond, cyclization can produce five‐ or six‐membered cycloalkanes (Path I‐iii). Five‐membered cycloalkanes can transform into cycloolefins via dehydrogenation (Path I‐i), while six‐membered ones lead to aromatics via dehydroaromatization (Path I‐vi). In Path II, the carbenium ion facilitates β‐scission, producing short‐chain hydrocarbons, including olefins and alkanes (Path II‐viii). Additionally, polymer chains may undergo direct hydrogenolysis by H_2_ or species from the dehydrogenation processes (Path III‐vii). External H_2_ promotes hydrogenolysis and hydrogenation but inhibits initial H_2_‐producing reactions, such as ring closure and dehydroaromatization. Notably, decreasing the Si/Al ratio of HZSM‐5 facilitates HDPE deconstruction due to the increased availability of Brønsted acid sites, which in turn promotes carbenium ion formation, cyclization, and β‐scission. While Ru/USY and Ru/SAPO‐34 have more acid sites, their steady‐state activities are significantly lower than Ru/HZSM‐5. This suggests that the constrained pore volume of HZSM‐5 prevents the formation and spread of fused aromatic rings, reducing coking and maintaining catalyst stability during HDPE upcycling.

Wang et al. noted that by integrating Pt into ZSM‐5, the BTX yield increased to 52% with a 31% yield of C_2_–C_4_ hydrocarbons.^[^ ^131^ ^]^ This surpassed the results from direct pyrolysis using ZSM‐5, which predominantly produced 66% of C_1_–C_4_ alkenes/alkanes and 21% BTX. Given dehydrogenation was proposed to be the rate‐determining step during the alkene aromatization on zeolites,^[^ ^132^ ^]^ the discrepancy likely arises from the distinct characteristics of sites for β‐hydrogen abstraction in Pt/ZSM‐5 compared to ZSM‐5. The DFT calculation showed that adsorbed ethene undergoes two consecutive oligomerization reactions to form alkyl fragments of Si‐OH***C_6_H_11_ and Pt‐H***C_6_H_11_ over ZSM‐5 and Pt/ZSM‐5, respectively.^[^ ^131^ ^]^ These fragments subsequently undergo cyclization to produce cyclic C_6_ species followed by dehydrogenation. Based on the identified transition states, β‐H abstractions required energy barriers of 2.79 eV (269 kJ mol^−1^) on ZSM‐5 and 1.39 eV(134 kJ mol^−1^) on Pt‐ZSM‐5. The final product, benzene, was produced after successive dehydrogenation processes.

**Figure 14:**
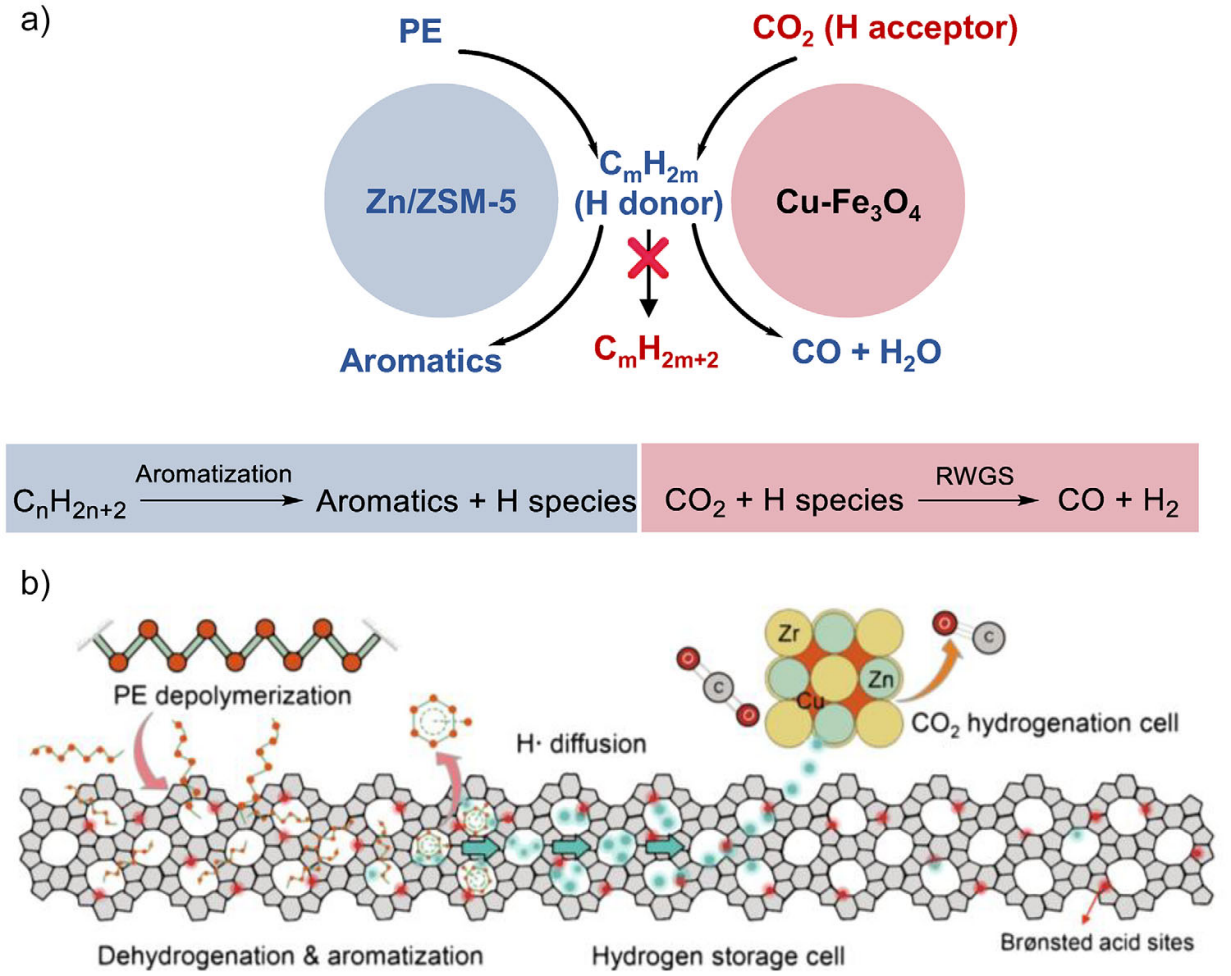
a) A coupling reaction of PE and CO_2_ to aromatics over Cu‐Fe_3_O_4_ and Zn/ZSM‐5 via tandem PE aromatization and CO_2_ hydrogenation. Reprinted with permission from Ref. [133]. Copyright 2023, Chinese Chemical Society. b) The proposed hydrogen capture and CO_2_ hydrogenation mechanism for PE‐CO_2_ co‐conversion in the presence of Brønsted acid sites of HZSM‐5. Reprinted with permission from Ref. [134]. Copyright 2024, AAAS.

It is important to note that the tandem hydrogenolysis/aromatization generates substantial amounts of H_2_, making C─C bond hydrogenolysis more favorable than aromatization. As a result, achieving aromatic selectivity greater than 50% in hydrogenolysis‐aromatization systems poses a significant challenge. Several studies have reported the introduction of CO_2_ as a hydrogen scavenger (via CO_2_ hydrogenation), effectively preventing alkane formation via hydrogenolysis and facilitating aromatization.^[^ ^133^, ^134^, ^135^ ^]^ For instance, Chen et al. utilized a physically mixed Cu‐Fe_3_O_4_ and Zn/ZSM‐5 as multifunctional catalysts, which significantly enhanced aromatic selectivity to 64.0% in the presence of 30 bar CO_2_ at 390 °C (Figure 14a).^[^ ^133^ ^]^ This approach creates tandem catalysis involving polyethylene aromatization and CO_2_ hydrogenation via reverse water‐gas shift reaction (RWGS, CO_2_ + H_2_→CO + H_2_O). Coinciding with this work, the CO_2_‐mediated PE aromatization was also investigated by Liu et al., who employed a combination of HZSM‐5 and CuZnZrO*_x_* catalysts.^[^ ^134^ ^]^ They achieved the highest aromatic yield of 62.5 wt% with a CO_2_ conversion of up to 1.5 mmol g_PE_^−1^ at 380 °C and 20 bar CO_2_ pressure. They posited that the Brønsted acid sites of HZSM‐5 function as a “hydrogen storage cell” (Figure 14b). H_2_ is generated from the cracking and aromatization of polyethylene over these Brønsted acid sites. It then diffuses through the sites to the adjacent CuZnZrO*_x_*. There, H_2_ is captured in situ by chemisorbed CO_2_, which is subsequently hydrogenated to CO.

Since both C─C bond cleavage and RWGS reactions are endothermic, they typically require operating temperatures exceeding 350 °C and high CO_2_ pressure to achieve satisfactory results. Note that CO_2_ does not participate in the cyclization/aromatization; instead, it is primarily converted into CO via RWGS. Inspired by the CO hydrogenation to aromatics in the oxide‐zeolite (OXZEO) catalysis, Ding et al. employed a bifunctional Pt/MnO*_x_*‐ZSM‐5 catalyst capable of converting polyolefins and CO_2_ below 300 °C.^[^ ^135^ ^]^ They achieved a yield as high as 64%, with BTX accounting for 60% of the aromatics produced. Intriguingly, CO_2_ not only acts as a hydrogen scavenger that suppresses alkane formation but also serves as a carbon source incorporated into the aromatics.

### 3.4 Metathesis

Alkane metathesis, achieved via the tandem processes of alkane dehydrogenation and olefin metathesis, is garnering increased attention in petrochemistry and organic synthesis.^[^ ^136^, ^137^ ^]^ This approach enables the interconversion between long‐chain and short‐chain alkanes, integrating alkane dehydrogenation, olefin metathesis, and alkene hydrogenation. Specifically, it is initiated by converting large paraffins into olefins, either via metal‐catalyzed dehydrogenation or through transfer dehydrogenation with smaller alkenes.^[^ ^138^ ^]^ Goldman et al. used Pincer‐ligated iridium (Ir) complexes to convert saturated alkanes into internal olefins and H_2_; these internal olefinic intermediates subsequently underwent cross‐metathesis with molybdenum alkylidene or supported Re_2_O_7_/Al_2_O_3_ catalysts to afford a range of olefin chain lengths, followed by rehydrogenation on the Ir catalyst to yield midrange paraffins.^[^ ^45^ ^]^ Given that the polyolefin is essentially an ultra‐long alkane, it is conceivable that kinetic coupling C─C cleavage with sequential metathesis enables low‐temperature conversions of polyolefins to a controlled alkane size.

**Figure 15:**
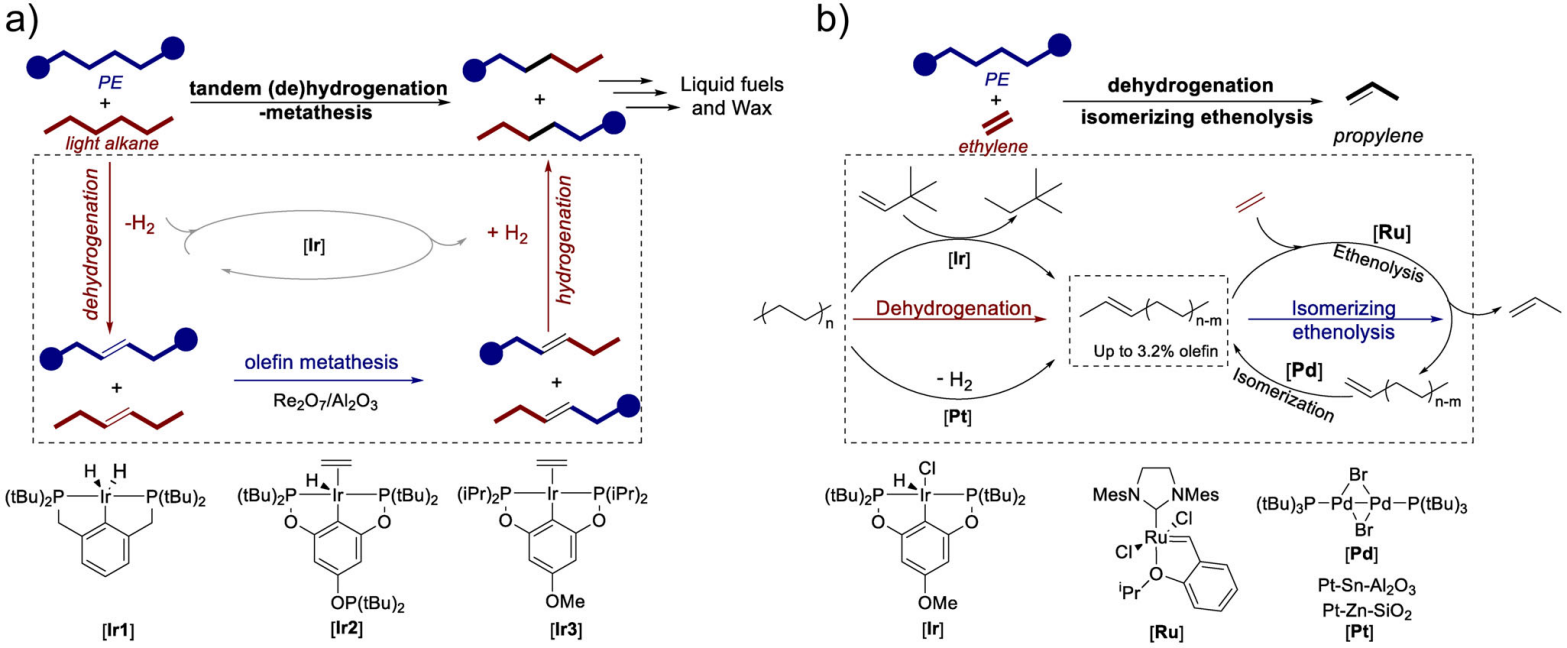
a) Coupling catalytic (de)hydrogenation and metathesis for converting PE to liquid fuels and waxes in the presence of a light alkane solvent (for example, *n*‐hexane). In this example, a molecular pincer [Ir] complex dehydrogenates saturated hydrocarbons to generate internal olefins and H_2_. Cross‐metathesis of the resulting olefins occurs over a supported ReO*_x_* catalyst; the resulting mid‐size olefins are re‐hydrogenated to generate mid‐size paraffins. Reprinted with permission from Ref. [33]. Copyright 2016, AAAS. b) Integrating partial dehydrogenation of PE and tandem isomerizing ethenolysis of the desaturated chain of PE into propylene. It is noteworthy that PE dehydrogenation can proceed via two distinct methods: either through a homogeneous transfer dehydrogenation of PE with *tert*‐butyl ethylene utilizing an [Ir] catalyst and NaOtBu in a p‐xylene solvent at 200 °C, or by means of heterogeneous dehydrogenation using a supported [Pt] catalyst under an argon flow at 350 °C. Reprinted with permission from Ref. [35]. Copyright 2022, AAAS.

Jia et al. employed a tandem cross‐alkane metathesis between polyolefin and light alkanes, such as *n*‐hexane and petroleum ether, achieving complete conversion of polyolefin into liquid fuels and waxes (ranging from C_7_ to C_38_) at 175 °C within 1–4 days, highlighting the low reaction rates as one of the challenges.^[^ ^33^ ^]^ Their dual‐catalyst system combined active pincer‐type Ir complexes for the dehydrogenation of PE and light alkane (as depicted in Figure 15a). The generated internal olefins underwent Re_2_O_7_/γ‐Al_2_O_3_‐catalyzed cross metathesis, leading to intermolecular mutual exchange of alkylidene fragments between two olefins. These were subsequently hydrogenated by the Ir catalyst into saturated alkanes. Importantly, the successful degradation of PE depended on the metathesis of its internal double bonds, controlled by the Ir complex, which in turn influenced the yields and distribution of the resulting liquid products. Bis(phosphinite)‐based Ir‐POCOP complexes ([Ir2] and [Ir3], as shown at the bottom of Figure 15a) exhibited superior performance compared to the bis(phosphine)‐based Ir‐PCP complex ([Ir1]). This reduced efficacy is attributed to complexes [Ir1] favoring the production of terminal olefins.

Despite the high regioselectivity of [Ir]‐type dehydrogenation catalysts for α‐olefin production, isomerization can convert terminal olefins into internal olefins. Kinetic coupling of olefin metathesis and isomerization enables the selective conversion of polyolefins into monomers.^[^ ^139^, ^140^ ^]^ In particular, employing ethylene (C_2_^=^) as the cross‐coupling agent could theoretically result in the sole formation of propylene (C_3_^=^), regardless of the position of the double bond or the length of the PE chain.^[^ ^141^ ^]^ Tandem isomerizing ethenolysis (I/E) is thermodynamically favored, with an estimated reaction standard free energy of approximately −6 kJ mol^−1^. Conk et al. developed a promising approach that integrates dehydrogenation with isomerizing olefin metathesis for upcycling of waste PE into monomer (Figure 15b).^[^ ^35^, ^142^ ^]^ Initial PE dehydrogenation can proceed via two distinct methods: either through a homogeneous transfer dehydrogenation of PE with *tert*‐butyl ethylene catalyzed by an [Ir] catalyst and NaOtBu in a p‐xylene solvent at 200 °C, or by means of heterogeneous dehydrogenation using a supported [Pt] catalyst under an argon flow at 350 °C.^[^ ^143^ ^]^ These methods resulted in an unsaturated PE primarily featuring internal olefins with degrees of unsaturation ranging from 0.6% to 3.2%. Following the formation of unsaturated polyethylene, the reaction mixture undergoes a tandem ethenolysis‐isomerization process with ethylene (C_2_^=^, 25 bar). This process is catalyzed by a combination of the 2^nd^ generation Hoveyda–Grubbs [Ru] metathesis catalyst and a dimeric [Pd] isomerization co‐catalyst, resulting in the production of propylene C_3_^=^ with yields exceeding 80%. Later, Coates and his coworkers used the [Ir2] complex for dehydrogenation, introducing unsaturation into HDPE.^[^ ^36^ ^]^ This was followed by a separate olefin metathesis step with 2‐hydroxyethyl acrylate, catalyzed by the 2^nd^ generation Hoveyda–Grubbs [Ru] catalyst, producing telechelic macromonomers, i.e., oligomers that have reactive functional groups at both ends of the molecular chain. These macromonomers were then repolymerized via transesterification, resulting in a polymer exhibiting mechanical properties analogous to the post‐consumer HDPE waste.

While molecular catalysts, especially iridium pincer complexes, are extensively employed to catalyze dehydrogenation, alkane metathesis and isomerization, their high cost, limited thermal stability, and the complexities involved in their separation from hydrocarbon mixtures pose significant challenges.^[^ ^144^, ^145^ ^]^ Wang et al. reported that methyltrioxorhenium supported on chlorinated alumina (CH_3_‐ReO_3_/Cl‐Al_2_O_3_) yields high selectivity for C_3_^=^ (∼95%) from monounsaturated PE via a tandem ethenolysis/isomerization process, comparable to the combination of homogeneous [Ru] metathesis catalyst and a dimeric [Pd] isomerization catalyst.^[^ ^34^ ^]^ Moreover, they observed that under batch conditions, C_3_^=^ selectivity decreases with higher conversion rates due to the formation of equilibrium olefin mixtures. Conversely, a semicontinuous process maintains high propylene selectivity (≥94%) by continuously removing propylene from the reaction mixture.

It should be noted that ReO*_x_* is notably costly and unsuitable for high‐temperature reactions due to the volatility of surface rhenium species; moreover, it poses challenges in terms of catalyst regeneration. Alternatively, Kim et al. have found that tungsten oxide supported on silica (WO*_x_*/SiO_2_) serves as an efficient catalyst for both olefin and alkane metathesis. The incorporation of zeolite 4A is essential to achieve high yields in these reactions, as it prevents the deactivation of the catalytic WO*_x_* species by oxygenates.^[^ ^146^ ^]^

**Figure 16:**
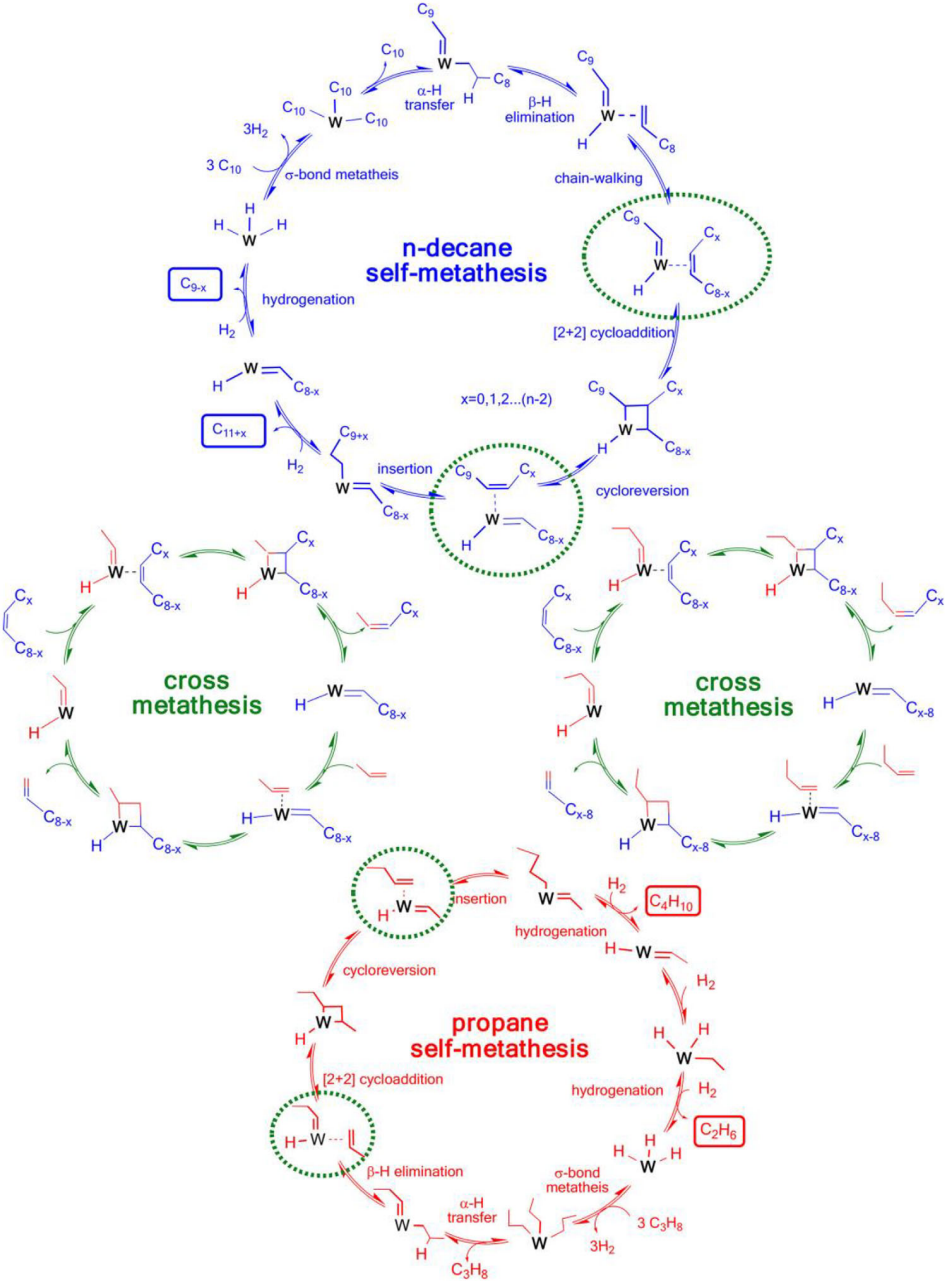
Proposed mechanism of alkane cross‐metathesis between *n*‐decane and propane. Reprinted with permission from Ref. [37]. Copyright 2019, American Chemical Society.

To elucidate the mechanistic details of cross‐metathesis between polyolefin and light paraffins, Basset and colleagues studied cross‐metathesis between *n*‐decane (*n*‐C_10_) and propane (C_3_) as well as self‐metathesis of each individual alkane using a well‐defined silica‐supported single‐site W catalyst, [(≡SiO)W(CH_3_)_2_(H)_3_].^[^ ^37^ ^]^ They found that the self‐metathesis of *n*‐C_10_ is approximately 8 times faster than that of C_3_. While *n*‐C_10_ produces alkanes spanning from C_2_ to C_19_, C_3_ predominantly produces C_2_ and C_4_. The efficiency of the cross‐metathesis between *n*‐C_10_ and C_3_ is governed by their molar ratio. At low C_3_/C_10_ molar ratios, the process is dominated by the self‐metathesis of *n*‐C_10_. A maximum around 50% of cross‐metathesis is observed at C_3_/C_10_ = 10. Any subsequent increase in this ratio does not enhance the cross‐metathesis percentage but rather directs the reaction toward C_3_ self‐metathesis. The proposed pathway, depicted in Figure 16, includes the self‐metathesis of C_3_ (red cycle) and *n*‐C_10_ (blue cycle) as well as various potential cross‐metathesis reactions (green cycle). The process begins with alkane dehydrogenation via C─H bond activation (σ‐bond metathesis), resulting in the generation of a metal alkyl, followed by the simultaneous formation of an alkene and metal hydride. Thereafter, olefin metathesis occurs, producing higher and lower olefins based on a metallocarbene created concurrently through α‐H abstraction from the metal alkyl. The coordinated olefin, resulting from the β‐H elimination, does not rapidly de‐coordinate from the W catalyst; instead, it undergoes a [2 + 2] cycloaddition with the coordinated carbene. Following the metallacycle cycloreversion, the newly formed olefin rapidly reacts with the coordinated hydride, leading to an alkyl group formation. This underscores the importance of maintaining a low steady‐state olefin concentration during alkane metathesis. The process then continues with the hydrogenation of these olefins on the metal hydride through an insertion‐elimination mechanism. Subsequently, olefin insertion is accompanied by the metal alkyl cleavage via hydrogen through σ‐bond metathesis. The carbene–hydride complex serves as the key intermediate in the mechanism, with the metal‐hydride performing dual roles: facilitating alkane dehydrogenation to olefins via C─H activation followed by β‐elimination, and hydrogenation. Meanwhile, the metal carbene and hydrogenation. Meanwhile, the metal carbene participates in the olefin metathesis through the traditional metallacyclobutane intermediate as described by the Chauvin mechanism.^[^ ^147^ ^]^

### 3.5 Oxidation

Aerobic oxidation of hydrocarbons is a frequently used strategy to incorporate oxygen‐containing functional groups into hydrocarbons, resulting in the production of diverse chemicals including alcohols, aldehydes, ketones, epoxides, and carboxylic acids.^[^ ^148^ ^]^ Direct upgrading of polyolefin waste with molecular oxygen at low temperatures is a “greener” alternative for producing valuable oxygen‐functionalized chemicals.^[^ ^149^ ^]^ Most polymers undergo slow autoxidative degradation in air, a process accelerated by UV light from the sun, which generates oxygen radicals. This results in the formation of hydroperoxides, carbonyl compounds, and carboxylic acid derivatives on the polymer surface.^[^ ^44^ ^]^ Hartwig et al. effectively employed a ruthenium‐oxo catalyst to oxidize LDPE, linear LDPE, and HDPE with high turnovers exceeding 2600 and achieving oxidation levels of up to 4 mol% along the polymer chain.^[^ ^150^ ^]^ Instead of using a homogeneous Ru catalyst combined with 2,6‐dichloropyridine N‐oxide as an oxidant, De Vos and coworkers used a layered titanosilicate catalyst (Ti‐ITQ‐6).^[^ ^151^ ^]^ This catalyst demonstrated high activity in the oxidation of PE using *tert*‐butyl hydroperoxide at temperatures below 100 °C, resulting in ketone‐functionalized PE with up to 3.4% functionalized carbon atoms. Furthermore, ketone‐functionalized PEs can be converted into ester‐functionalized PEs via Baeyer‐Villiger oxidation of ketones. Alternatively, these ketones can also react with hydroxylamine, followed by a liquid‐phase Beckmann rearrangement, to produce oxime‐functionalized and amide‐functionalized PEs.

Partenheimer reported that polyolefins undergo aerobic oxidation to form fatty acids using catalyst systems such as V/Br in water or Co/Mn/Br in acetic acid. Notably, polypropylene was converted to acetic acid with a 63% yield, while polyethylene yielded a 47% mixture of succinic, glutaric, and adipic acids, both under conditions of 150–180 °C and 70 bar air within several hours.^[^ ^152^ ^]^ The oxidation pathway proceeds as follows: Br acts as a promoter, initiating hydrogen abstraction from the hydrocarbon to produce bromide. Mn^3+^ oxidizes the bromide ion to form Mn^2+^. Then, Co^3+^ re‐oxidizes Mn^2+^ to Mn^3+,^ leading to the formation of Co^2+^. Finally, Co^2+^ is further oxidized back to Co^3+^ via peroxide decomposition.^[^ ^44^ ^]^ Nevertheless, the use of corrosive Br as a vital catalyst component in this process, along with the production of undesirable byproducts like methyl bromide, renders the process environmentally unsustainable.^[^ ^153^, ^154^ ^]^ Alternatively, carbon radical generation from hydrocarbons can be realized through the utilization of a phthalimide N‐oxyl (PINO) radical, which can be generated from *N*‐hydroxyphthalimide (NHPI) with O_2_ under mild conditions (below 100 °C).^[^ ^155^ ^]^

**Figure 17:**
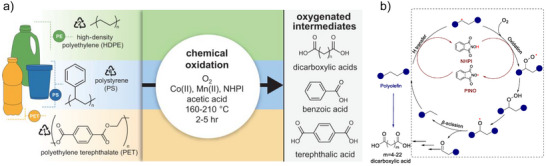
a) Upcycling of mixed plastic waste vi oxidation. b) Reaction pathways of tandem oxidation‐cracking for converting polyolefin to fatty acids. Reprinted with permission from Ref. [38]. Copyright 2022, AAAS.

Sullivan et al. employed this oxidation strategy to depolymerize mixed polymers, including polyolefins, into a mixture of oxygenated small molecules. These molecules serve as advanced feedstock for biological conversion (Figure 17a).^[^ ^38^ ^]^ The reaction initiates with the in situ decomposition of NHPI into PINO radicals using Co and Mn catalysts (Figure 17b). Following this, hydrogen atoms are transferred from the C─H bonds within the polymer backbone, resulting in the formation of alkyl radicals (R•) and the regeneration of NHPI. Subsequently, these R• radicals react with O_2_ to produce peroxide radicals (RO_2_•), which then decompose into corresponding alkoxyl radicals (RO•). These RO• radicals can undergo C─C cleavage to form aldehydes, ketones, and chain terminates via β‐scission steps. The repeated cycle of hydrogen atom transfer, aerobic oxidation, and C─C cleavage ultimately results in the formation of a mixture of low‐molecular‐weight fatty acids.

However, free radical oxidation via NHPI requires high catalyst loadings of homogenous Co/Mn compounds, making this process potentially unsustainable. In a recent study by Wang et al., a heterogeneous catalyst, Ru/TiO_2_, was used to convert almost all the LDPE into liquid products under a pressure of 15 bar with air at 160 °C for 24 h.^[^ ^39^ ^]^ Control experiments showed that conducting the reaction under an N_2_ atmosphere, instead of air, resulted in no liquid product formation, highlighting the involvement of oxygen in the catalytic reaction. When TiO_2_ was used alone, it yielded 50% solid residue and approximately 40% adipic acid. In comparison, TiO_2‐_supported Ru significantly boosted the oil yield to 85%, with minimal solid residue, and the resulting oil product was confirmed to be carboxylic acid. The cooperation of Ru and TiO_2_ efficiently promotes the oxidative upcycling reactions of PE. Zhang et al. found that adjusting the cobalt loading of MCM‐41 allows for controllable distribution of dicarboxylic acids within 24 h under a pressure of 15 bar of air at 160 °C. This distribution ranges from short‐chain (C_4_–C_10_) to long‐chain (C_10_–C_20_), dicarboxylic acids, which is attributed to the confinement effect of the pore channels and the dispersion of cobalt.^[^ ^156^ ^]^

Li and coworkers reported a multistage strategy in which the pyrolysis of polyolefins generates an oil, followed by hydroformylation and hydrogenation of the olefin products, resulting in paraffins, aromatics, mono‐, and dialcohols in the range of C_5_–C_20_.^[^ ^157^ ^]^ Initially, they conducted direct pyrolysis of waste polyolefins to produce pyrolysis oils with high concentrations of olefins (>50 wt%) in a fluidized bed reactor at 500 °C and a residence time of 20 seconds. This was followed by hydroformylation of the pyrolysis oil at 120 °C and 70 bar of synthesis gas (CO/H_2_ = 1) using a commercial Co_2_(CO)_8_ catalyst. More than 90% of the olefins in the pyrolysis oil were converted into aldehydes, achieving a yield of up to 60 wt%. While the aldehydes can be hydrogenated into alcohols, however, it requires the removal of a significant amount of homogeneous cobalt catalyst (with the cobalt catalyst accounting for 10 wt% relative to the pyrolysis oil). Meanwhile, Xu et al. reported a catalyst‐ and hydrogen‐free temperature‐gradient thermolysis strategy that utilizes precise temperature control at 360 °C during heating.^[^ ^158^ ^]^ This approach enables the controlled degradation of PE and PP into waxes while inhibiting the production of small molecules. The waxes formed were subsequently oxidized on Mn catalysts to yield fatty acids with number‐average molar masses of approximately 700 and 670 Da. However, the degradation products consist of long‐chain hydrocarbons (typically >C_20_) with broad distributions. Furthermore, selectively controlling the production of terminal alkenyl intermediates remains challenging. Most recently, Munyaneza et al. used a custom‐designed thermolysis reactor that can control the thermolysis temperature gradient using cooling design, effectively shifting the product hydrocarbon distribution from wax to alkene‐rich oil with high α‐olefin contents.^[^ ^159^ ^]^ The alkene‐rich oil is further upgraded into sulfate detergents via H_2_SO_4_ treatment.

## 4 Conclusions and Outlook

The energy‐efficient upcycling of discarded polyolefins represents a pivotal step toward achieving a circular economy; however, the kinetic and thermodynamic stability of polyolefins makes their conversion a formidable challenge. To address the thermodynamic limitations, recent strategies focus on the kinetic coupling of endothermic C─C bond cleavage with exothermic reactions that redistribute H‐atoms and form new C─C bonds. This coupling is achieved by integrating primary C─C bond cleavage with processes such as alkylation, hydrogenolysis, hydrocracking, metathesis, cyclization, and oxidation, as summarized in Table 1. These coupled reactions enable simultaneous molecular size alteration and utilization of exothermic pathways, yielding a diverse array of products, including monomers, fuels, lubricants, and components for advanced polymers.

**Table 1:** Summary of advances in catalytic upcycling of polyolefins via kinetic coupling of the endothermic C─C bond cleavage of polyolefins with reactions including alkylation, hydrogenolysis, hydrocracking, metathesis, cyclization/aromatization, and oxidation.

Integrated Strategy	Catalyst	Polyolefin Type	Co‐reactant	Temperature	Pressure	Time	Main Products	Reference
Alkylation	Chloroaluminate ionic liquid ([C_4_Py]Cl‐2AlCl_3_)	LDPE/PP	iC_4_/iC_5_	70 °C	1 bar	3 h	Liquid isoalkanes	[40, 71]
	Anhydrous AlCl_3_	LDPE	iC_5_	60 °C	1 bar	30 min	Liquid isoalkanes	[72]
Hydrogenolysis	Pt/SrTiO_3_	PE	H_2_	300 °C	11.7 bar	96 h	Lubricants and waxes	[26]
	mSiO_2_/Pt/SiO_2_	HDPE	H_2_	250 °C	13.8 bar	6 h	Lubricants	[87]
	Ru/C	LDPE/PP	H_2_	250 °C	20 bar	16 h	Liquid alkanes	[19]
	Ru/TiO_2_	PP	H_2_	250 °C	30 bar	16 h	Lubricants	[23, 94]
	Ru/CeO_2_	LDPE	H_2_	240 °C	60 bar	8 h	Liquid alkanes	[95]
	Zr(neopentyl)_2_/sulfated alumina	LDPE/PP	H_2_	190 °C	2 bar	48 min	Light hydrocarbons	[101]
	Co/SiO_2_	LDPE	H_2_	275 °C	30 bar	8 h	Liquid alkanes	[102]
	Ni/SiO_2_	LDPE	H_2_	280 °C	20 bar	9 h	Liquid alkanes	[103]
	NiAl‐T	LDPE/PP	H_2_	350 °C	30 bar	2–14 h	Liquid *n*‐alkanes	[104]
Hydrocracking	Ru/H‐BEA	LDPE/PP	H_2_	215 °C	30 bar	16 h	Liquid isoalkanes	[28]
	Ru/WO_3_/ZrO_2_	LDPE	H_2_	250 °C	50 bar	2–16 h	Liquid alkanes and wax	[20, 24]
	2D Pt/WO_3_	HDPE	H_2_	250 °C	30 bar	3 h	Liquid alkanes	[29]
	Pt/WO_3_/ZrO_2_	LDPE/HDPE	H_2_	250 °C	30 bar	2–24 h	Liquid isoalkanes	[113, 114]
	Pt/WO_3_/ZrO_2_ + HY	LDPE	H_2_	300 °C	30 bar	2 h	Liquid isoalkanes	[27]
	Pt/5Ce‐HY	LDPE	H_2_	300 °C	30 bar	2 h	Liquid isoalkanes	[116]
	Ni/HZSM‐5 Co/HZSM‐5	LDPE	H_2_	375 °C	45 bar	16 h	Liquid isoalkanes	[121]
	MoS*_x_*‐Hbeta	LDPE	H_2_	300 °C	30 bar	16 h	Liquid isoalkanes	[122]
Cyclization/Aromatization	Pt/γAl_2_O_3_	LDPE/HDPE	−	280 °C	1 bar	24 h	Long‐chain alkylaromatics	[30]
	Pt/F‐Al_2_O_3_ Pt/Cl‐Al_2_O_3_	LDPE/HDPE	−	280 °C	1 bar	8 h	Long‐chain alkylaromatics	[11]
	Ru/HZSM‐5 (Si/Al = 300)	HDPE	−	280 °C	1 bar	24 h	Cycloalkanes/aromatics	[31]
	Cu‐Fe_3_O_4_ + Zn/ZSM‐5	LDPE	CO_2_	390 °C	30 bar	1 h	Aromatics	[133]
	CuZnZrO*_x_* + HZSM‐5	LDPE	CO_2_	380 °C	20 bar	200 min	Aromatics	[134]
	Pt/MnO*_x_*‐ZSM‐5	LDPE	CO_2_	280 °C	10 bar	3 h	Aromatics	[135]
Metathesis	Pincer‐type Ir complexes + Re_2_O_7_/γ‐Al_2_O_3_	HDPE	*n*‐C_6_	150 °C	1 bar	3 days	Liquid alkanes and waxes	[33]
	[Ir‐^tBu^POCOP + *tert*‐butyl Ethylene + Nao NaOtBu]+[Ru] +[Pd]	HDPE	C_2_H_4_	200 + 130 °C	25 bar	12 h + 16 h	Propylene	[35]
	WO_3_/SiO_2_ + Na/γAl_2_O_3_	PE/PP	C_2_H_4_	320 °C	15 bar	90 min	Propylene/isobutylene	[142]
	CH_3_‐ReO_3_/Cl‐Al_2_O_3_	PE	C_2_H_4_	100 °C	1 bar	1 h	Propylene	[34]
Oxidation	Polyfluorinated ruthenium porphyrin	LDPE	2,6‐dichloropyridine N‐oxide	120 °C	1 bar	2 h	Oxidized PE	[150]
	Layered titanosilicate (TiITQ‐6)	PE	*tert*‐butyl hydroperoxide	100 °C	1 bar	6–24 h	Ketone‐functionalized PE	[151]
	Co(OAc)_2_ + Mn(OAc)_2_ + Zr(acac)_4_ + N‐hydroxyphthalimide	HDPE, PS, PET	O_2_	160–210 °C	1 bar	2–5 h	Carboxylic acids	[38]
	CoCl_2_·6H_2_O + MnSO_4_·H_2_O + HBr	PE, PS, PP	O_2_	120 °C	1 bar	48 h	Carboxylic acids	[149]
	Ru/TiO_2_	LDPE	O_2_	160 °C	15 bar	24 h	Carboxylic acids	[39]
	Co‐MCM‐41	PE	O_2_	125 °C	10 bar	24 h	Carboxylic acids	[156]

Thermodynamic coupling, while essential, is insufficient for efficient polymer conversion at low temperatures. Catalysts must be designed to achieve high reactivity, and in the case of cooperative catalysis, the distance between catalytic sites, as well as their abundance and strength, are critical parameters. Consequently, a comprehensive understanding of the elementary steps is indispensable. This is particularly challenging for polymers due to the complexity of their interactions and mobility on the catalyst surface. Additionally, the impact of solvents and catalyst interactions on the thermodynamic state of the polymer and its products will significantly influence both reactivity and selectivity.

The coupling of alkylation with primary C─C cleavage marks a significant achievement as it represents the first controlled conversion of polyolefins to alkanes at temperatures below 100 °C. While it is established that these reactions involve carbenium ion intermediates, achieving a molecular‐level comprehension of the properties and reactivity of charged and uncharged reactants and intermediates in this specific environment calls for further insights. These include (i) understanding the characteristics of the polar solvent environment, (ii) elucidating interactions with solids such as polymers and acid/metal catalyst components, and (iii) gaining a deeper understanding of the elementary steps involved in polyolefin transformations and intermediate formation. The primary objective of future research is to establish a comprehensive understanding of the catalysts, the role played by the solvent, and the interconnected kinetics governing low‐temperature cracking and alkylation in the presence of ionic liquids. This effort aims to produce alkanes with precisely controlled molecular weight and branching degree.

Hydrocracking, involving the addition of hydrogen to products resulting from Brønsted acid‐catalyzed C─C bond cleavage, offers a practical means to balance the endothermic nature of the cleavage step. This process necessitates the cooperative action of metal and acid sites to catalyze both C─C cleavage and hydrogen addition, as well as hydrogen abstractions from reactants. Understanding the effective interplay between these distinct catalytic sites and the coupling of acid‐catalyzed C─C bond manipulation with metal‐catalyzed hydrogen addition remains the critical goal. To facilitate this interplay between sites, various approaches can be employed, including physical mixtures, intimate mixtures, metal nanoparticles on solid acid surfaces, and encapsulation within the mesopores of hierarchical zeolites. Solvent interactions also play a crucial role in controlling this communication. The relative rates of individual catalytic functions and their proximity raise fundamental questions that must be addressed through reaction‐diffusion formalisms. The primary objective of future research is to optimize the spatial arrangement of distinct catalytic functions and enhance their interplay. This approach aims to improve and balance the rates of intramolecular hydride shifts and intermolecular hydride transfers. Additionally, employing polar solvents in combination with metal nanoparticles and co‐fed hydrogen is expected to increase the rates of hydrogen addition and abstraction, enabling precise control over the number of subsequent internal hydride shift events at the acid sites. Employing tailored hierarchically structured or nano‐sized zeolites will provide the means to fine‐tune the separation of cooperating functions, as well as variations in their strengths and concentrations.

Cyclization and intermolecular rearrangements, coupled with metal‐catalyzed C─C cleavage in polyolefins, can yield valuable aromatics and surfactants even without H_2_. The bifunctional metal‐acid catalysts enable thermodynamic and kinetic coupling through hydrogen redistribution at relatively low temperatures. Metal sites initiate the dehydrogenation of polyolefin, forming C═C bonds, while the Brønsted acid sites convert these intermediates into carbenium ions. However, interactions between metal surfaces and polyolefin chains are more intricate than with small hydrocarbons, owing to multiple adsorption possibilities arising from branching and entanglement. This complexity may explain the positive effect of smaller particle sizes on the intrinsic rates of dehydrogenation and hydrogenolysis. The precise role of the acid sites in the tandem mechanism governs the extent of ring closure and dehydroaromatization steps necessary for the dehydrocyclization of intermediates resulting from the initial hydrogenolysis. Therefore, adjusting acid strength, acid site density, and pore diameter, as well as the tailoring customization of active site properties, is crucial to control reaction rates for C‐C bond cleavage, isomerization, hydrogen addition and abstraction, and the extent of branching and product distribution in alkylaromatics.

Combining C‐C bond cleavage with olefin metathesis allows for a reduction in the required reaction temperature while enabling the production of olefin monomers and alkanes as intermediates. It involves sequential alkane dehydrogenation, thermoneutral alkene metathesis, and isomerization reactions, each with established precedents but lacking a precise mechanistic understanding required for effective coupling. While reaction parameters and the content ratio of bifunctional sites have been extensively studied for alkenes, the same needs to be determined for the coupling of C─C cleavage and metal‐catalyzed dehydrogenation of polyolefins with metathesis. Thus, understanding the mechanisms and controlling the relative rates of C─C cleavage, dehydrogenation, and metathesis, as well as their compatibility for converting polyolefins at low temperatures and with feasible rates, remains a challenge. Additionally, identifying the activation barriers for element‐abundant metal catalysts will provide insights into strategies for achieving activation at milder temperatures, complementing the use of noble metal catalysts.

Finally, the upcycling of polyolefin waste into valuable aliphatic dicarboxylic acids through aerobic oxidation is an attractive and energy‐efficient process for niche applications that eliminates the need for hydrogen. This approach enhances economic viability and reduces dependence on fossil resources. However, recent processes that rely on oxidation via a radical addition mechanism involving epoxy intermediate rearrangements suffer from poor selectivity. The C─H bonds in the resulting alcohol and ketone products are weaker than those in the hydrocarbons, making them susceptible to hydrogen abstraction reactions and undesirable radical reactions. To address this challenge, catalyst improvements are required to selectively introduce oxygen functionality at regular intervals along the polymer chain at mild conditions.

## Conflict of Interests

The authors declare no conflict of interest.

## Data Availability

The data that support the findings of this study are available from the corresponding author upon reasonable request.

## References

[1] J. M. Garcia , M. L. Robertson , Science 2017, 358, 870–872. 29146798 10.1126/science.aaq0324

[2] J. Wassenaar , Qenos White Paper – Circular Polyolefin Capacity Set to Reach 1 Million Tonnes Globally in 2025 , 2023.

[3] Polyolefins Global Market Report 2024, https://www.researchandmarkets.com/reports/5741672/polyolefins‐global‐market‐report (accessed: February 2025).

[4] R. Geyer , J. R. Jambeck , K. L. Law , Sci. Adv. 2017, 3, e1700782. 28776036 10.1126/sciadv.1700782 PMC5517107

[5] R. Geyer , in Plastic Waste and Recycling (Ed.: T. M. Letcher ), Academic Press, 2020, pp. 13–32. 10.1016/B978-0-12-817880-5.00002-5.

[6] J. M. García , Chem 2016, 1, 813–815.

[7] R. C. Thompson , W. Courtene‐Jones , J. Boucher , S. Pahl , K. Raubenheimer , A. A. Koelmans , Science 2024, 386, eadl2746. 39298564 10.1126/science.adl2746

[8] B. D. Vogt , K. K. Stokes , S. K. Kumar , ACS Appl. Polym. Mater. 2021, 3, 4325–4346.

[9] M. He , K. Zhang , Y. Guan , Y. Sun , B. Han , Natl. Sci. Rev. 2023, 10, nwad046. 37565189 10.1093/nsr/nwad046 PMC10411673

[10] Refinery Capacity Report, 2022, https://www.eia.gov/petroleum/refinerycapacity/ (accessed: January 2024).

[11] J. Sun , Y.‐H. Lee , R. D. Yappert , A. M. LaPointe , G. W. Coates , B. Peters , M. M. Abu‐Omar , S. L. Scott , Chem 2023, 9, 2318–2336.

[12] J.‐P. Lange , ACS Sustain. Chem. Eng. 2021, 9, 15722–15738. 10.1021/acssuschemeng.1c02114 PMC8411582 34484990

[13] D. P. Serrano , J. Aguado , J. M. Escola , ACS Catal. 2012, 2, 1924–1941.

[14] T. Tan , W. Wang , K. Zhang , Z. Zhan , W. Deng , Q. Zhang , Y. Wang , ChemSusChem 2022, 15, e202200522. 35438240 10.1002/cssc.202200522

[15] L. D. Ellis , N. A. Rorrer , K. P. Sullivan , M. Otto , J. E. McGeehan , Y. Román‐Leshkov , N. Wierckx , G. T. Beckham , Nat. Catal. 2021, 4, 539–556.

[16] I. Vollmer , M. J. F. Jenks , M. C. P. Roelands , R. J. White , T. van Harmelen , P. de Wild , G. P. van der Laan , F. Meirer , J. T. F. Keurentjes , B. M. Weckhuysen , Angew. Chem. Int. Ed. 2020, 59, 15402–15423. 10.1002/anie.201915651 PMC7497176 32160372

[17] I. de Marco , B. M. Caballero , A. López , M. F. Laresgoiti , A. Torres , M. J. Chomón , J. Anal. Appl. Pyrolysis 2009, 85, 384–391.

[18] S. M. Al‐Salem , A. Antelava , A. Constantinou , G. Manos , A. Dutta , J. Environ. Manage. 2017, 197, 177–198. 28384612 10.1016/j.jenvman.2017.03.084

[19] J. E. Rorrer , G. T. Beckham , Y. Román‐Leshkov , JACS Au 2021, 1, 8–12. 34467267 10.1021/jacsau.0c00041 PMC8395642

[20] C. Wang , T. Xie , P. A. Kots , B. C. Vance , K. Yu , P. Kumar , J. Fu , S. Liu , G. Tsilomelekis , E. A. Stach , W. Zheng , D. G. Vlachos , JACS Au 2021, 1, 1422–1434. 34604852 10.1021/jacsau.1c00200 PMC8479762

[21] C. Jia , S. Xie , W. Zhang , N. N. Intan , J. Sampath , J. Pfaendtner , H. Lin , Chem. Catal. 2021, 1, 437–455.

[22] L. Chen , L. C. Meyer , L. Kovarik , D. Meira , X. I. Pereira‐Hernandez , H. Shi , K. Khivantsev , O. Y. Gutiérrez , J. Szanyi , ACS Catal. 2022, 12, 4618–4627.

[23] P. A. Kots , T. Xie , B. C. Vance , C. M. Quinn , M. D. de Mello , J. A. Boscoboinik , C. Wang , P. Kumar , E. A. Stach , N. S. Marinkovic , L. Ma , S. N. Ehrlich , D. G. Vlachos , Nat. Commun. 2022, 13, 5186. 36057603 10.1038/s41467-022-32934-5 PMC9440920

[24] C. Wang , K. Yu , B. Sheludko , T. Xie , P. A. Kots , B. C. Vance , P. Kumar , E. A. Stach , W. Zheng , D. G. Vlachos , Appl. Catal., B 2022, 319, 121899.

[25] G. Zichittella , A. M. Ebrahim , J. Zhu , A. E. Brenner , G. Drake , G. T. Beckham , S. R. Bare , J. E. Rorrer , Y. Román‐Leshkov , JACS Au 2022, 2, 2259–2268. 36311830 10.1021/jacsau.2c00402 PMC9597591

[26] G. Celik , R. M. Kennedy , R. A. Hackler , M. Ferrandon , A. Tennakoon , S. Patnaik , A. M. LaPointe , S. C. Ammal , A. Heyden , F. A. Perras , M. Pruski , S. L. Scott , K. R. Poeppelmeier , A. D. Sadow , M. Delferro , ACS Cent. Sci. 2019, 5, 1795–1803. 31807681 10.1021/acscentsci.9b00722 PMC6891864

[27] S. Liu , P. A. Kots , B. C. Vance , A. Danielson , D. G. Vlachos , Sci. Adv. 2021, 7, eabf8283. 33883142 10.1126/sciadv.abf8283 PMC11426200

[28] J. E. Rorrer , A. M. Ebrahim , Y. Questell‐Santiago , J. Zhu , C. Troyano‐Valls , A. S. Asundi , A. E. Brenner , S. R. Bare , C. J. Tassone , G. T. Beckham , Y. Román‐Leshkov , ACS Catal. 2022, 12, 13969–13979.

[29] Q. Zhou , D. Wang , Q. Wang , K. He , K. H. Lim , X. Yang , W.‐J. Wang , B.‐G. Li , P. Liu , Angew. Chem. Int. Ed. 2023, 62, e202305644. 10.1002/anie.202305644 37325872

[30] F. Zhang , M. Zeng , R. D. Yappert , J. Sun , Y.‐H. Lee , A. M. LaPointe , B. Peters , M. M. Abu‐Omar , S. L. Scott , Science 2020, 370, 437–441. 33093105 10.1126/science.abc5441

[31] J. Du , L. Zeng , T. Yan , C. Wang , M. Wang , L. Luo , W. Wu , Z. Peng , H. Li , J. Zeng , Nat. Nanotechnol. 2023, 18, 772–779. 37365277 10.1038/s41565-023-01429-9

[32] J. H. Miller , A. K. Starace , D. A. Ruddy , ChemSusChem 2022, 15, e202200535. 35395145 10.1002/cssc.202200535

[33] X. Jia , C. Qin , T. Friedberger , Z. Guan , Z. Huang , Sci. Adv. 2016, 2, e1501591. 27386559 10.1126/sciadv.1501591 PMC4928905

[34] N. M. Wang , G. Strong , V. DaSilva , L. Gao , R. Huacuja , I. A. Konstantinov , M. S. Rosen , A. J. Nett , S. Ewart , R. Geyer , S. L. Scott , D. Guironnet , J. Am. Chem. Soc. 2022, 144, 18526–18531. 36178850 10.1021/jacs.2c07781

[35] R. J. Conk , S. Hanna , J. X. Shi , J. Yang , N. R. Ciccia , L. Qi , B. J. Bloomer , S. Heuvel , T. Wills , J. Su , A. T. Bell , J. F. Hartwig , Science 2022, 377, 1561–1566. 36173865 10.1126/science.add1088 PMC11723507

[36] A. Arroyave , S. Cui , J. C. Lopez , A. L. Kocen , A. M. LaPointe , M. Delferro , G. W. Coates , J. Am. Chem. Soc. 2022, 144, 23280–23285. 36524740 10.1021/jacs.2c11949

[37] N. Morlanés , S. G. Kavitake , D. C. Rosenfeld , J.‐M. Basset , ACS Catal. 2019, 9, 1274–1282.

[38] K. P. Sullivan , A. Z. Werner , K. J. Ramirez , L. D. Ellis , J. R. Bussard , B. A. Black , D. G. Brandner , F. Bratti , B. L. Buss , X. Dong , S. J. Haugen , M. A. Ingraham , M. O. Konev , W. E. Michener , J. Miscall , I. Pardo , S. P. Woodworth , A. M. Guss , Y. Román‐Leshkov , S. S. Stahl , G. T. Beckham , Science 2022, 378, 207–211. 36227984 10.1126/science.abo4626

[39] K. Wang , R. Jia , P. Cheng , L. Shi , X. Wang , L. Huang , Angew. Chem. Int. Ed. 2023, 62, e202301340. 10.1002/anie.202301340 37211533

[40] W. Zhang , S. Kim , L. Wahl , R. Khare , L. Hale , J. Hu , D. M. Camaioni , O. Y. Gutiérrez , Y. Liu , J. A. Lercher , Science 2023, 379, 807–811. 36821681 10.1126/science.ade7485

[41] R. Zhao , G. L. Haller , J. A. Lercher , Microporous Mesoporous Mater. 2023, 358, 112390.

[42] C. A. Gärtner , A. C. van Veen , J. A. Lercher , J. Am. Chem. Soc. 2014, 136, 12691–12701. 25118821 10.1021/ja505411s

[43] A. Feller , I. Zuazo , A. Guzman , J. O. Barth , J. A. Lercher , J. Catal. 2003, 216, 313–323.

[44] W. Partenheimer , Catal. Today 1995, 23, 69–158.

[45] A. S. Goldman , A. H. Roy , Z. Huang , R. Ahuja , W. Schinski , M. Brookhart , Science 2006, 312, 257–261. 16614220 10.1126/science.1123787

[46] D. W. Flaherty , E. Iglesia , J. Am. Chem. Soc. 2013, 135, 18586–18599. 24266427 10.1021/ja4093743

[47] S. Yue , P. Wang , B. Yu , T. Zhang , Z. Zhao , Y. Li , S. Zhan , Adv. Energy Mater. 2023, 13, 2302008.

[48] J. Wei , J. Liu , W. Zeng , Z. Dong , J. Song , S. Liu , G. Liu , Catal. Sci. Technol. 2023, 13, 1258–1280.

[49] F. Eisenreich , Angew. Chem. Int. Ed. 2023, 62, e202301303. 10.1002/anie.202301303 37051840

[50] H. Zhou , Y. Wang , Y. Ren , Z. Li , X. Kong , M. Shao , H. Duan , ACS Catal. 2022, 12, 9307–9324.

[51] R.‐X. Yang , K. Jan , C.‐T. Chen , W.‐T. Chen , K. C. W. Wu , ChemSusChem 2022, 15, e202200171. 35349769 10.1002/cssc.202200171

[52] M. Chu , Y. Liu , X. Lou , Q. Zhang , J. Chen , ACS Catal. 2022, 12, 4659–4679.

[53] S. S. Borkar , R. Helmer , F. Mahnaz , W. Majzoub , W. Mahmoud , M. m. Al‐Rawashdeh , M. Shetty , Chem. Catal. 2022, 2, 3320–3356.

[54] J. Payne , M. D. Jones , ChemSusChem 2021, 14, 4041–4070. 33826253 10.1002/cssc.202100400 PMC8518041

[55] S. C. Kosloski‐Oh , Z. A. Wood , Y. Manjarrez , J. P. de los Rios , M. E. Fieser , Mater. Horiz. 2021, 8, 1084–1129. 34821907 10.1039/d0mh01286f

[56] X. Jiao , K. Zheng , Z. Hu , S. Zhu , Y. Sun , Y. Xie , Adv. Mater. 2021, 33, 2005192. 10.1002/adma.202005192 33834571

[57] X. Chen , Y. Wang , L. Zhang , ChemSusChem 2021, 14, 4137–4151. 34003585 10.1002/cssc.202100868

[58] A. J. Martín , C. Mondelli , S. D. Jaydev , J. Pérez‐Ramírez , Chem 2021, 7, 1487–1533.

[59] H. Chen , K. Wan , Y. Zhang , Y. Wang , ChemSusChem 2021, 14, 4123–4136. 33998153 10.1002/cssc.202100652

[60] H. Lv , F. Huang , F. Zhang , Langmuir 2024, 40, 5077–5089. 38358312 10.1021/acs.langmuir.3c03866

[61] C. W. S. Yeung , J. Y. Q. Teo , X. J. Loh , J. Y. C. Lim , ACS Mater. Lett. 2021, 3, 1660–1676.

[62] M. Chu , W. Tu , S. Yang , C. Zhang , Q. Li , Q. Zhang , J. Chen , SusMat 2022, 2, 161–185.

[63] K. Faust , P. Denifl , M. Hapke , ChemCatChem 2023, 15, e202300310.

[64] L. Gan , Z. Dong , H. Xu , H. Lv , G. Liu , F. Zhang , Z. Huang , CCS Chem. 2024, 6, 313–333.

[65] S. Xu , J. Tang , L. Fu , Langmuir 2024, 40, 3984–4000. 38364857 10.1021/acs.langmuir.3c03195

[66] W. Kaminsky , I.‐J. N. Zorriqueta , J. Anal. Appl. Pyrolysis 2007, 79, 368–374.

[67] A. Feller , J. A. Lercher , in Advances in Catalysis, Vol. 48, Academic Press, 2004, pp. 229–295, 10.1016/s0360-0564(04)48003-1.

[68] Refinery Alkylation Units Capacity and Capital Expenditure Outlook and Forecast with Details of All Operating and Planned Alkylation Units to 2028, GlobalData, 2024, https://www.globaldata.com/store/report/oil‐and‐gas‐refinery‐alkylation‐units‐market‐analysis/ (accessed: January 2024).

[69] H. K. Timken , H. Luo , B.‐K. Chang , E. Carter , M. Cole , in Commercial Applications of Ionic Liquids (Ed.: M. B. Shiflett ), Springer International Publishing, Cham 2020, pp. 33–47.

[70] H. K. C. Timken , S. Elomari , S. Trumbull , R. Cleverdon , U.S. Patent 7,432,408, 2008.

[71] W. Zhang , R. Khare , S. Kim , L. Hale , W. Hu , C. Yuan , Y. Sheng , P. Zhang , L. Wahl , J. Mai , B. Yang , O. Y. Gutiérrez , D. Ray , J. Fulton , D. M. Camaioni , J. Hu , H. Wang , M.‐S. Lee , J. A. Lercher , Nat. Commun. 2024, 15, 5785. 38987244 10.1038/s41467-024-49827-4 PMC11237162

[72] W. Zhang , H. Yao , R. Khare , P. Zhang , B. Yang , W. Hu , D. Ray , J. Hu , D. M. Camaioni , H. Wang , S. Kim , M.‐S. Lee , M. L. Sarazen , J. G. Chen , J. Lercher , Angew. Chem. Int. Ed. 2024, 63, e202319580. 10.1002/anie.202319580 38433092

[73] P. A. Kots , B. C. Vance , D. G. Vlachos , React. Chem. Eng. 2022, 7, 41–54.

[74] B. Hernández , P. Kots , E. Selvam , D. G. Vlachos , M. G. Ierapetritou , ACS Sustain. Chem. Eng. 2023, 11, 7170–7181.

[75] J. Weitkamp , ChemCatChem 2012, 4, 292–306.

[76] G. C. Bond , Metal‐Catalysed Reactions of Hydrocarbons, Vol. 2, Springer, US, 2005, 10.1007/b136857.

[77] J. G. Speight , in Kirk‐Othmer Encyclopedia of Chemical Technology, Portico, 2018, pp. 1–46, 10.1002/0471238961.1805060919160509.a01.pub3.

[78] K. L. Sánchez‐Rivera , G. W. Huber , ACS Cent. Sci. 2021, 7, 17–19. 33532565 10.1021/acscentsci.0c01637 PMC7844849

[79] D. W. Flaherty , D. D. Hibbitts , E. I. Gürbüz , E. Iglesia , J. Catal. 2014, 311, 350–356.

[80] D. W. Flaherty , A. Uzun , E. Iglesia , J. Phys. Chem. C 2015, 119, 2597–2613.

[81] D. W. Flaherty , D. D. Hibbitts , E. Iglesia , J. Am. Chem. Soc. 2014, 136, 9664–9676. 24961991 10.1021/ja5037429

[82] D. D. Hibbitts , D. W. Flaherty , E. Iglesia , J. Phys. Chem. C 2016, 120, 8125–8138.

[83] J. Kim , S. Sun , D. Kim , B. G. Park , H. Lee , W. Huang , K. An , Chem Catal. 2024, 4, 101076.

[84] Y.‐Y. Wang , A. Tennakoon , X. Wu , C. Sahasrabudhe , L. Qi , B. G. Peters , A. D. Sadow , W. Huang , ACS Catal. 2024, 14, 2084–2094.

[85] F. Regali , R. S. París , A. Aho , M. Boutonnet , S. Järås , Top. Catal. 2013, 56, 594–601.

[86] S. D. Jaydev , A. J. Martín , J. Pérez‐Ramírez , ChemSusChem 2021, 14, 5179–5185. 34553832 10.1002/cssc.202101999

[87] A. Tennakoon , X. Wu , A. L. Paterson , S. Patnaik , Y. Pei , A. M. LaPointe , S. C. Ammal , R. A. Hackler , A. Heyden , I. I. Slowing , G. W. Coates , M. Delferro , B. Peters , W. Huang , A. D. Sadow , F. A. Perras , Nat. Catal. 2020, 3, 893–901.

[88] X. Wu , A. Tennakoon , R. Yappert , M. Esveld , M. S. Ferrandon , R. A. Hackler , A. M. LaPointe , A. Heyden , M. Delferro , B. Peters , A. D. Sadow , W. Huang , J. Am. Chem. Soc. 2022, 144, 5323–5334. 35195400 10.1021/jacs.1c11694

[89] P. Hu , C. Zhang , M. Chu , X. Wang , L. Wang , Y. Li , T. Yan , L. Zhang , Z. Ding , M. Cao , P. Xu , Y. Li , Y. Cui , Q. Zhang , J. Chen , L. Chi , J. Am. Chem. Soc. 2024, 146, 7076–7087. 38428949 10.1021/jacs.4c00757

[90] G. C. Bond , R. R. Rajaram , R. Burch , J. Phys. Chem. 1986, 90, 4877–4881.

[91] G. C. Bond , X. Yide , J. Chem. Soc., Chem. Commun. 1983, 21, 1248–1249.

[92] Y. Nakagawa , S.‐i. Oya , D. Kanno , Y. Nakaji , M. Tamura , K. Tomishige , ChemSusChem 2017, 10, 189–198. 27863013 10.1002/cssc.201601204

[93] M. Tamura , S. Miyaoka , Y. Nakaji , M. Tanji , S. Kumagai , Y. Nakagawa , T. Yoshioka , K. Tomishige , Appl. Catal., B 2022, 318, 121870.

[94] P. A. Kots , S. Liu , B. C. Vance , C. Wang , J. D. Sheehan , D. G. Vlachos , ACS Catal. 2021, 11, 8104–8115.

[95] Y. Nakaji , M. Tamura , S. Miyaoka , S. Kumagai , M. Tanji , Y. Nakagawa , T. Yoshioka , K. Tomishige , Appl. Catal., B 2021, 285, 119805.

[96] L. Chen , Y. Zhu , L. C. Meyer , L. V. Hale , T. T. Le , A. Karkamkar , J. A. Lercher , O. Y. Gutiérrez , J. Szanyi , React. Chem. Eng. 2022, 7, 844–854.

[97] H. Ji , X. Wang , X. Wei , Y. Peng , S. Zhang , S. Song , H. Zhang , Small, 2023, 19, e2300903. 37096905 10.1002/smll.202300903

[98] Z. Zhang , J. Wang , X. Ge , S. Wang , A. Li , R. Li , J. Shen , X. Liang , T. Gan , X. Han , X. Zheng , X. Duan , D. Wang , J. Jiang , Y. Li , J. Am. Chem. Soc. 2023, 145, 22836–22844. 37794780 10.1021/jacs.3c09338

[99] S. Lu , Y. Jing , S. Jia , M. Shakouri , Y. Hu , X. Liu , Y. Guo , Y. Wang , ChemCatChem 2023, 15, e202201375.

[100] V. Dufaud , J.‐M. Basset , Angew. Chem. Int. Ed. 1998, 37, 806–810. 10.1002/(SICI)1521-3773(19980403)37:6<806::AID-ANIE806>3.0.CO;2-6 29711396

[101] A. H. Mason , A. Motta , A. Das , Q. Ma , M. J. Bedzyk , Y. Kratish , T. J. Marks , Nat. Commun. 2022, 13, 7187. 36418305 10.1038/s41467-022-34707-6 PMC9684440

[102] S. S. Borkar , R. Helmer , S. Panicker , M. Shetty , ACS Sustain. Chem. Eng. 2023, 11, 10142–10157.

[103] B. C. Vance , P. A. Kots , C. Wang , J. E. Granite , D. G. Vlachos , Appl. Catal., B 2023, 322, 122138.

[104] B. C. Vance , S. Najmi , P. A. Kots , C. Wang , S. Jeon , E. A. Stach , D. N. Zakharov , N. Marinkovic , S. N. Ehrlich , L. Ma , D. G. Vlachos , JACS Au 2023, 3, 2156–2165. 37654574 10.1021/jacsau.3c00232 PMC10466342

[105] M. J. Sterba , V. Haensel , Ind. Eng. Chem. Prod. Res. Dev. 1976, 15, 2–17.

[106] W. Ding , J. Liang , L. L. Anderson , Energy Fuels 1997, 11, 1219–1224.

[107] S. Uçar , S. Karagöz , T. Karayildirim , J. Yanik , Polym. Degrad. Stab. 2002, 75, 161–171.

[108] D. Munir , M. F. Irfan , M. R. Usman , Renew. Sustain. Energy Rev. 2018, 90, 490–515.

[109] S. Kokuryo , K. Tamura , S. Tsubota , K. Miyake , Y. Uchida , A. Mizusawa , T. Kubo , N. Nishiyama , ChemCatChem 2023, 15, e202300461.

[110] T. Kwon , B. Ahn , K. H. Kang , W. Won , I. Ro , Nat. Commun. 2024, 15, 10239. 39613753 10.1038/s41467-024-54495-5 PMC11607347

[111] J. Shang , Y. Li , Y. Hu , T. Zhang , T. Wang , J. Zhang , H. Yan , Y. Liu , X. Chen , X. Feng , X. Zhang , C. Yang , D. Chen , J. Catal. 2024, 430, 115302.

[112] W.‐T. Lee , F. D. Bobbink , A. P. van Muyden , K.‐H. Lin , C. Corminboeuf , R. R. Zamani , P. J. Dyson , Cell Rep. Phys. Sci. 2021, 2, 100332.

[113] B. C. Vance , P. A. Kots , C. Wang , Z. R. Hinton , C. M. Quinn , T. H. Epps , L. T. J. Korley , D. G. Vlachos , Appl. Catal., B 2021, 299, 120483.

[114] Z. R. Hinton , P. A. Kots , M. Soukaseum , B. C. Vance , D. G. Vlachos , T. H. Epps , L. T. J. Korley , Green Chem. 2022, 24, 7332–7339.

[115] P. A. Kots , P. A. Doika , B. C. Vance , S. Najmi , D. G. Vlachos , ACS Sustain. Chem. Eng. 2023, 11, 9000–9009.

[116] X. Wu , X. Wang , L. Zhang , X. Wang , S. Song , H. Zhang , Angew. Chem. Int. Ed. 2024, 63, e202317594. 10.1002/anie.202317594 38183405

[117] J. Duan , W. Chen , C. Wang , L. Wang , Z. Liu , X. Yi , W. Fang , H. Wang , H. Wei , S. Xu , Y. Yang , Q. Yang , Z. Bao , Z. Zhang , Q. Ren , H. Zhou , X. Qin , A. Zheng , F.‐S. Xiao , J. Am. Chem. Soc. 2022, 144, 14269–14277. 35914188 10.1021/jacs.2c05125

[118] J. Z. Tan , C. W. Hullfish , Y. Zheng , B. E. Koel , M. L. Sarazen , Appl. Catal., B 2023, 338, 123028.

[119] J. Z. Tan , M. Ortega , S. A. Miller , C. W. Hullfish , H. Kim , S. Kim , W. Hu , J. Z. Hu , J. A. Lercher , B. E. Koel , M. L. Sarazen , ACS Catal. 2024, 14, 7536–7552.

[120] L. Chen , J. B. Moreira , L. C. Meyer , J. Szanyi , Appl. Catal., B 2023, 335, 122897.

[121] W.‐T. Lee , A. van Muyden , F. D. Bobbink , M. D. Mensi , J. R. Carullo , P. J. Dyson , Nat. Commun. 2022, 13, 4850. 35977921 10.1038/s41467-022-32563-y PMC9385622

[122] Z. Qiu , S. Lin , Z. Chen , A. Chen , Y. Zhou , X. Cao , Y. Wang , B.‐L. Lin , Sci. Adv. 2023, 9, eadg5332. 37343106 10.1126/sciadv.adg5332 PMC10284556

[123] Z. Cen , X. Han , L. Lin , S. Yang , W. Han , W. Wen , W. Yuan , M. Dong , Z. Ma , F. Li , Y. Ke , J. Dong , J. Zhang , S. Liu , J. Li , Q. Li , N. Wu , J. Xiang , H. Wu , L. Cai , Y. Hou , Y. Cheng , L. L. Daemen , A. J. Ramirez‐Cuesta , P. Ferrer , D. C. Grinter , G. Held , Y. Liu , B. Han , Nat. Chem. 2024, 16, 871–880. 38594366 10.1038/s41557-024-01506-z PMC11164678

[124] W. Han , L. Lin , Z. Cen , Y. Ke , Q. Xu , J. Zhu , X. Mei , Z. Xia , X. Zheng , Y. Wang , Y. Liu , M. He , H. Wu , B. Han , Chem 2025, 11, 102340. 10.1002/anie.202417923 39537576

[125] W. Han , L. Lin , Z. Cen , Y. Ke , Q. Xu , J. Zhu , X. Mei , Z. Xia , X. Zheng , Y. Wang , Y. Liu , M. He , H. Wu , B. Han , Angew. Chem. Int. Ed. 2025, 64, e202417923. 10.1002/anie.202417923 39537576

[126] J. N. Hancock , J. E. Rorrer , Appl. Catal., B 2023, 338, 123071.

[127] Z. Zhang , H. Chen , G. Li , W. Hu , B. Niu , D. Long , Y. Zhang , ACS Catal. 2024, 14, 2552–2561.

[128] Y. Uemichi , K. Takuma , A. Ayame , Chem. Commun. 1998, 18, 1975–1976.

[129] M. L. Pennel , A. K. Maurya , A. M. Ebrahim , C. J. Tassone , M. Cargnello , ACS Sustain. Chem. Eng. 2023, 11, 12623–12630.

[130] C.‐F. Chang , S. Rangarajan , J. Phys. Chem. A 2023, 127, 2958–2966. 36975726 10.1021/acs.jpca.3c01444 PMC10249406

[131] W. Wang , C. Yao , X. Ge , X. Pu , J. Yuan , W. Sun , W. Chen , X. Feng , G. Qian , X. Duan , Y. Cao , Z. Yang , X. Zhou , J. Zhang , J. Mater. Chem. A 2023, 11, 14933–14940.

[132] C. Tu , H. Fan , D. Wang , N. Rui , Y. Du , S. D. Senanayake , Z. Xie , X. Nie , J. G. Chen , Appl. Catal., B 2022, 304, 120956.

[133] W. Chen , Y. Jiao , Y. Liu , M. Wang , F. Zhang , D. Ma , CCS Chem. 2024, 6, 1422–1429.

[134] Y. Liu , B. Ma , J. Tian , C. Zhao , Sci. Adv. 2024, 10, eadn0252. 38608025 10.1126/sciadv.adn0252 PMC11014447

[135] Y. Ding , S. Zhang , C. Liu , Y. Shao , X. Pan , X. Bao , Natl. Sci. Rev. 2024, 11, nwae097. 38660412 10.1093/nsr/nwae097 PMC11042496

[136] V. Vidal , A. Théolier , J. Thivolle‐Cazat , J.‐M. Basset , Science 1997, 276, 99–102. 9082995 10.1126/science.276.5309.99

[137] G. C. Vougioukalakis , R. H. Grubbs , Chem. Rev. 2010, 110, 1746–1787. 20000700 10.1021/cr9002424

[138] J.‐M. Basset , C. Coperet , D. Soulivong , M. Taoufik , J. T. Cazat , Acc. Chem. Res. 2010, 43, 323–334. 19856892 10.1021/ar900203a

[139] D. Guironnet , B. Peters , J. Phys. Chem. A 2020, 124, 3935–3942. 32310647 10.1021/acs.jpca.0c01363

[140] G. W. Coates , Y. D. Y. L. Getzler , Nat. Rev. Mater. 2020, 5, 501–516.

[141] M. Nagyházi , Á. Lukács , G. Turczel , J. Hancsók , J. Valyon , A. Bényei , S. Kéki , R. Tuba , Angew. Chem. Int. Ed. 2022, 61, e202204413. 10.1002/anie.202204413 PMC9400880 35420225

[142] R. J. Conk , J. F. Stahler , J. X. Shi , J. Yang , N. G. Lefton , J. N. Brunn , A. T. Bell , J. F. Hartwig , Science 2024, 0, eadq7316. 10.1126/science.adq7316 39208080

[143] L. D. Ellis , S. V. Orski , G. A. Kenlaw , A. G. Norman , K. L. Beers , Y. Román‐Leshkov , G. T. Beckham , ACS Sustain. Chem. Eng. 2021, 9, 623–628. 38706722 10.1021/acssuschemeng.0c07612 PMC11066966

[144] I. Göttker‐Schnetmann , P. White , M. Brookhart , J. Am. Chem. Soc. 2004, 126, 1804–1811. 14871112 10.1021/ja0385235

[145] X. Zhang , S.‐B. Wu , X. Leng , L. W. Chung , G. Liu , Z. Huang , ACS Catal. 2020, 10, 6475–6487.

[146] D. Kim , Z. R. Hinton , P. Bai , L. T. J. Korley , T. H. Epps , R. F. Lobo , Appl. Catal., B 2022, 318, 121873.

[147] P. Jean‐Louis Hérisson , Y. Chauvin , Die Makromolekulare Chemie 1971, 141, 161–176.

[148] F. Cavani , J. H. Teles , ChemSusChem 2009, 2, 508–534. 19536755 10.1002/cssc.200900020

[149] B. Zhao , H. Tan , J. Yang , X. Zhang , Z. Yu , H. Sun , J. Wei , X. Zhao , Y. Zhang , L. Chen , D. Yang , J. Deng , Y. Fu , Z. Huang , N. Jiao , The Innovation 2024, 5, 100586. 38414518 10.1016/j.xinn.2024.100586 PMC10897897

[150] L. Chen , K. G. Malollari , A. Uliana , D. Sanchez , P. B. Messersmith , J. F. Hartwig , Chem 2021, 7, 137–145.

[151] R. Lemmens , J. Vercammen , L. Van Belleghem , D. De Vos , Nat. Commun. 2024, 15, 9188. 39448613 10.1038/s41467-024-53506-9 PMC11502679

[152] W. Partenheimer , Catal. Today 2003, 81, 117–135.

[153] R. A. F. Tomás , J. C. M. Bordado , J. F. P. Gomes , Chem. Rev. 2013, 113, 7421–7469. 23767849 10.1021/cr300298j

[154] L. Chen , K. G. Malollari , A. Uliana , J. F. Hartwig , J. Am. Chem. Soc. 2021, 143, 4531–4535. 33734671 10.1021/jacs.1c00125

[155] Y. Ishii , S. Sakaguchi , Catal. Today 2006, 117, 105–113.

[156] Q. Zhang , J. He , X. Wei , C. Shen , P. Ye , W. An , X. Liu , H. Li , S. Xu , Z. Su , Y.‐Z. Wang , Angew. Chem. Int. Ed. 2024, 63, e202407510. 10.1002/anie.202407510 38774971

[157] H. Li , J. Wu , Z. Jiang , J. Ma , V. M. Zavala , C. R. Landis , M. Mavrikakis , G. W. Huber , Science 2023, 381, 660–666. 37561862 10.1126/science.adh1853

[158] Z. Xu , N. E. Munyaneza , Q. Zhang , M. Sun , C. Posada , P. Venturo , N. A. Rorrer , J. Miscall , B. G. Sumpter , G. Liu , Science 2023, 381, 666–671. 37561876 10.1126/science.adh0993

[159] N. E. Munyaneza , R. Ji , A. DiMarco , J. Miscall , L. Stanley , N. Rorrer , R. Qiao , G. Liu , Nat. Sustain. 2024, 7, 1681–1690.

